# Superheating in mafic magmas controls clinopyroxene nucleation delay and magma ascent dynamics

**DOI:** 10.1038/s41467-026-73352-1

**Published:** 2026-06-08

**Authors:** Barbara Bonechi, Fabio Arzilli, Margherita Polacci, Alessandro Fabbrizio, Giuseppe La Spina, Eleni Michailidou, Elisa Biagioli, Richard A. Brooker, Jean-Louis Hazemann, Robert C. Atwood, Danilo Di Genova, Sumith Abeykoon, David A. Neave, Renat R. Almeev, Mike Burton

**Affiliations:** 1 Department of Earth and Environmental Sciences, The University of Manchester, Manchester, UK; 2 School of Science and Technology, Geology Division, University of Camerino, Camerino, Italy; 3 Dipartimento di Scienze dell’Ambiente e della Terra - DISAT, Università degli Studi di Milano-Bicocca, Milano, Italy; 4 Istituto Nazionale di Geofisica e Vulcanologia (INGV), Sezione Osservatorio Etneo, Catania, Italy; 5 Institute of Petrology and Structural Geology, Faculty of Science, Charles University, Prague, Czech Republic; 6 School of Earth Sciences, University of Bristol, Bristol, UK; 7 Université Grenoble Alpes, CNRS, Grenoble INP, Institut Néel, Grenoble, France; 8 Diamond Light Source, Harwell Science and Innovation Campus, Harwell, UK; 9 Institute of Science, Technology and Sustainability for Ceramics (ISSMC), National Research Council (CNR), Faenza, Italy; 10 Institute of Earth System Sciences, Section of Mineralogy, Leibniz University Hannover, Hannover, Germany

**Keywords:** Volcanology, Petrology

## Abstract

Crystallisation kinetics play a fundamental role in controlling conduit dynamics and eruptive style. The degree of superheating is critical in controlling crystallisation kinetics; however, its effect is still debated and has an unclear impact on eruption dynamics. Here, we investigate how superheating influences clinopyroxene nucleation in tephritic magmas from the 2021 Tajogaite eruption (La Palma, Spain) through both in situ and ex situ view experiments. Our findings show that superheating delays nucleation by dissolving pre-existing nuclei, thereby inhibiting crystallisation upon return to subliquidus conditions. Using a numerical model, we investigate how different nucleation delays resulting from different degrees of superheating affect magma ascent dynamics. Depending on the initial thermodynamic conditions and on the pre-eruptive history of magma, an increased nucleation delay can significantly reduce crystal content during ascent, lowering magma viscosity and affecting eruptive style. These findings highlight the critical role of pre-eruptive thermal histories in controlling eruptive style, and provide constraints for refining experimental protocols and numerical models, with direct implications for improving volcanic hazard assessment and eruption forecasting.

## Abstract (web summary)

This study shows that heating magma above its crystallisation temperature delays crystal formation, reducing crystal content during ascent and controlling volcanic eruption style and intensity.

## Introduction

Forecasting the eruptive style of volcanoes and understanding its relationship with the physical state of magma reservoirs remains one of the foremost challenges in modern volcanology^1^. Volcanoes can erupt effusively or explosively, with transitions between these styles occurring either within a single eruption or between distinct events^2^. Understanding these variations is critical for volcanic risk assessment, as different eruptive styles pose widely different hazards^3^.

Recent studies have shown that the initial, pre-eruptive conditions of the magma reservoir influence syn-eruptive conduit processes^1,4–6^, playing key roles in changing eruptive styles. These conditions include factors such as reheating via primitive magma recharge^7–9^, the presence of exsolved volatile phases in the magma chamber^7,10^, volatile dilution^11^, nanoscale structure of the melt phase^12^, and varying crystal content prior to eruption^4–6,13^. Among these factors, the crystal content is particularly important because it exerts a first-order control on magma viscosity, thereby influencing degassing efficiency, magma ascent dynamics, and ultimately eruptive style. Crystal content depends on the residence times of magma within the chamber, the extent to which crystals are entrained by magma flow and accumulated within the chamber^14,15^, and delays in crystal nucleation associated with superheating relative to the liquidus temperature^16,17^. Such superheating may arise not only from magma recharge but also from decompression of water undersaturated magmas during ascent, which can produce effective superheating relative to the liquidus even in the absence of external heat input, as commonly observed in water undersaturated basaltic systems^18–20^.

Superheating influences crystal nucleation by imposing an “incubation period”^21^, a time delay required for the assembly of critical clusters at a given temperature^16^. However, the mechanisms underlying this effect remain debated. Two contrasting views have emerged regarding superheating’s impact on nucleation kinetics. Some studies suggest that the effect is negligible, arguing that silicate melt structure relaxes rapidly enough to become path-independent within the timescales (hours) of crystallisation experiments^22^. In contrast, others propose that superheating fundamentally alters melt structure, thereby extending nucleation delay^16,23,24^. The structural perspective offers a compelling mechanistic explanation. When crystals dissolve during superheating, the surrounding melt retains a “ghost” of the crystalline structure, a local ordering that persists even after the mineral disappears^17^. Higher superheating temperatures progressively disrupt the residual order^14,19,20^, ultimately producing a more homogeneous liquid by eliminating both sub-critical nuclei and compositional heterogeneities inherited from the starting material. This structural homogenisation requires greater undercooling to re-establish the thermodynamic driving force for nucleation^25,26^. The thermodynamic consequences of superheating extend beyond structural effects. Classical nucleation theory predicts that superheating increases both the critical nucleus size and the activation energy barrier for nucleation^27^. This occurs because the dissolution of pre-existing nuclei during superheating eliminates preferential nucleation sites, forcing subsequent crystallisation to proceed via homogeneous rather than heterogeneous mechanisms. Additionally, superheating influences nucleation through its effect on melt dynamics and element distribution. Enhanced thermal energy promotes cation diffusion and breaks down medium-range order in the melt network, creating a more statistically random distribution of network-forming and network-modifying cations^28^. This randomisation means that upon cooling, the system must overcome not only the thermodynamic barrier for nucleation but also the kinetic barriers associated with reorganising randomly distributed cations into ordered crystalline arrangements. These combined effects (namely, structural homogenisation, increased activation barriers, and cation redistribution) explain why superheated melts consistently show longer nucleation delays and require greater undercooling to initiate crystallisation compared to melts held near their liquidus temperatures^29^.

Nucleation represents the first step in the structural transformation of silicate melts, initiating the rearrangement of atoms from a disordered liquid into ordered crystalline clusters^28–30^. Critically, this process exhibits significant time delays in magmatic systems—stable nuclei do not form immediately upon reaching thermodynamically favourable conditions, but instead require incubation periods ranging from minutes to days^21,31–37^. Understanding these nucleation delays is essential for constraining magmatic timescales in both volcanic and plutonic systems. Previous investigations of nucleation kinetics have provided valuable but incomplete insights. Studies on basaltic systems have explored various aspects including texture development^35,38–40^, effects of cooling rate and supercooling^21,31,32^, and nucleation mechanisms^33,36^, revealing delay times from 30 min at high undercooling to hundreds of hours near the liquidus. Similar work on felsic compositions^34,37,41–48^ has yielded quantitative nucleation and growth rates, though nucleation delay was rarely the primary focus. Crucially, no study has systematically examined how varying degrees of superheating control nucleation delay times, nor linked these delays to eruptive dynamics through integrated experimental and numerical approaches.

Here, we address this gap by investigating superheating effects on clinopyroxene nucleation delay in tephritic magma from the 2021 Tajogaite eruption (La Palma, Spain). Our approach combines real-time crystallisation imaging via synchrotron X-ray microtomography with conventional experimental petrology, providing unprecedented temporal resolution of nucleation processes. Specifically, by adopting nucleation delays resulting from our experiments into a numerical model of magma ascent, we demonstrate how pre-eruptive thermal histories can control syn-eruptive crystallisation and, ultimately, volcanic eruption styles. This study does not aim to provide a comprehensive synthesis or reinterpretation of the Tajogaite eruption sequence, nor to quantitatively reconstruct its temporal evolution. Instead, we want to elucidate a general physical mechanism, i.e. the effect of superheating-induced nucleation delay on crystallinity, magma viscosity, and ascent dynamics, that may operate in mafic magmas more broadly. The 2021 Tajogaite magma is used as a case study because its tephritic-basanitic composition is well constrained and has been extensively characterised in previous studies^49–60^, allowing it to be employed as starting material in our experiments and enabling a direct link between experimental observations and natural magmatic conditions. In addition, Tajogaite represents a low-viscosity eruption involving hot, water-undersaturated magma rising from a deep source^50–52^. The high pre-eruptive temperatures inferred for this system^50,54^ suggest that the magma may have experienced some degree of superheating prior to eruption and during ascent, making it a suitable natural scenario in which to explore the potential effects of superheating on crystallisation dynamics.

## Results and discussion

### *Experimental design and setup for investigating superheating effects*

**Fig. 1:**
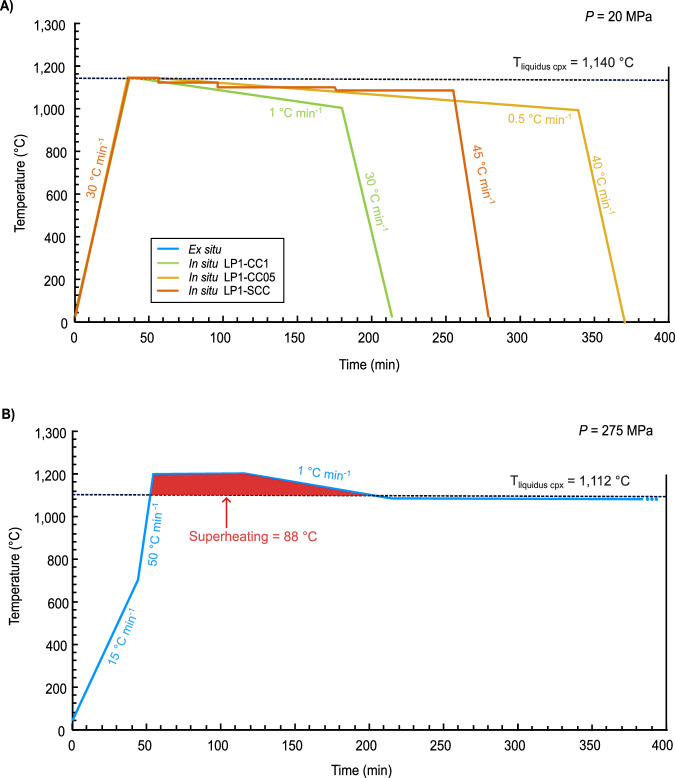
Experimental conditions for in situ and ex situ view experiments. Experimental conditions of (**A**) in situ and (**B**) ex situ view experiments reported as function of temperature and time. Dashed line shows clinopyroxene liquidus temperature calculated using Rhyolite-MELTS software^63,64^ and experimentally constrained. Clinopyroxene liquidus temperature is calculated for the tephrite with 1 wt.% H_2_O.

**Fig. 2:**
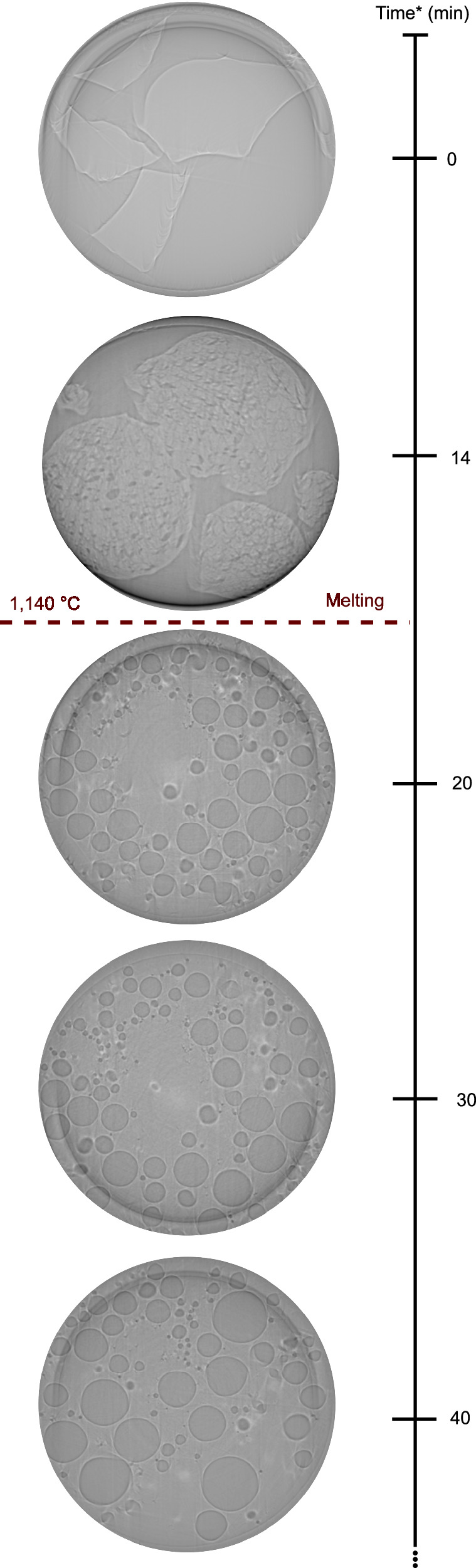
Time-resolved microtomographic slices depicting glass melting. Reconstructed slices show the melting of the glassy starting material, observed in real-time. Time* represents the elapsed time from the start of the microtomographic recording at ~960 °C. Melting occurs at ~1140 °C within the first 20 min of recording.

We performed a series of experiments to investigate how different degrees of superheating influence pre-eruptive crystallisation. We focused on clinopyroxene, the dominant crystallising phase in these magmas, which is readily detectable in both in situ and ex situ view experiments (see Methods). This makes it particularly suitable for quantitatively constraining crystallisation kinetics. The first set of experiments, with in situ view and no superheating (Δ*T*_sh_ = *T*-*T*_Lcpx_ = 0 °C, where *T* is the temperature of the experiment and *T*_Lcpx_ is the liquidus temperature of clinopyroxene), simulates a scenario where magma does not undergo superheating prior to eruption. Then magma is cooled down at a given cooling rate, resulting in a progressive increase in undercooling (Δ*T*_uc_ = *T*_Lcpx_−*T*) with time. This experimental approach reproduces the net increase in undercooling expected during magma ascent, which in natural systems may arise from a combination of non-adiabatic cooling and an increase in liquidus temperature associated with volatile exsolution (e.g., H_2_O loss) during decompression^18,61^. In contrast, the second set of experiments, with ex situ view (see Methods for details) and superheating (Δ*T*_sh_ = ~90 °C), simulates a scenario of substantial superheating in mafic magmas, which may occur naturally due to magma recharge or decompression during ascent. The system is then cooled at a constant rate down to the liquidus temperature, where it is held for the duration of the experiment prior to quenching. Experiments with in situ view were performed at beamline I12-JEEP, Diamond Light Source, Harwell (United Kingdom), combining a dedicated X-ray transparent Internally Heated Pressure Vessel apparatus^62^ with fast synchrotron X-ray microtomography, while ex situ view experiments were performed at the Experimental Petrology Laboratory of Charles University, Prague (Czech Republic), by using a Quick press non-end loaded piston-cylinder (for details see Methods). Ex situ view experiments were essential to access longer durations (8–20 h) that are not feasible at synchrotron facilities due to beamtime constraints. Experimental conditions are reported in Supplementary Data 1. The ex situ view experiments were performed at 275 MPa and 1200 °C (superliquidus) followed, after 1 h of dwell time, by a cooling step (1 °C min^−1^) to 1100 °C (Fig. 1). This dwell time at superliquidus temperature (Δ*T*_sh_ = ~90 °C) is one of the two main differences, the other being in the preparation of the starting material (see Methods). The in situ view experiments were performed at 20 MPa and 1140 °C (*T*_Lcpx_; Fig. 1). After maintaining the temperature constant for 20 min at *T*_Lcpx_ (Δ*T*_sh_ = 0 °C), the system was cooled at a rate of 1 °C min^−1^. The *T*_Lcpx_ at 20 MPa was calculated using Rhyolite-MELTS^63,64^ and validated visually during 4D in situ view experiments. From the microtomography data, we observed in real time that the structural relaxation of the starting material began at ~960 °C during the heating ramp (14 min after the start of the tomographic recording; Fig. 2). This relaxation corresponds to the glass passing its glass transition temperature (*T*_g_)^65^ and beginning to behave as a molten liquid, gradually losing its original shape. Complete melting of the starting material was reached at ~1140 °C (after 20 min; Fig. 2). This temperature, experimentally determined, closely matches the value estimated with Rhyolite-MELTS, supporting its use as a reasonable approximation of *T*_Lcpx_ under the investigated pressure conditions. The *T*_Lcpx_ at 275 MPa (~1112 °C; Fig. 1) was instead constrained through crystallisation experiments^49^.

### *Nucleation delay: role of pre-existing nuclei and superheating*

We compared the intermediate-pressure (20 MPa) experiments with in situ view (Δ*T*_sh_ = 0 °C; Δ*T*_uc_ = ~10–20 °C) with the ex situ view (Δ*T*_sh_ = ~90 °C; Δ*T*_uc_ = ~12 °C) ones at high-pressure (275 MPa). The main difference we observed between in situ and ex situ view experiments is a nucleation delay. In the experiments with ex situ view, clinopyroxene crystallisation only occurred after 8 h, while in the experiments with in situ view it occurs in the first 20 min of cooling at temperatures between 1140 and 1120 °C (Supplementary Fig. S1 and Supplementary Data 1). In their 4D synchrotron X-ray microtomography experiments at high temperature and atmospheric pressure, Polacci et al.^36^ observed a nucleation delay of 12 min for pyroxene at Δ*T*_uc_ = 38 °C and 30 min at Δ*T*_uc_ = 18 °C in a basaltic sample of the 2001 Mt. Etna eruption. Our in situ results align with their findings.

To elucidate the contrasting nucleation behaviours observed between in situ and ex situ view experiments, we systematically investigated two potential controlling factors: the presence of pre-existing nuclei and the influence of superheating on clinopyroxene nucleation kinetics. Pre-existing nuclei, which could originate from incomplete melting during sample preparation or form during experimental heating, were evaluated using complementary spectroscopic and thermal analytical techniques. Raman spectroscopy provided direct structural characterization of the starting glasses, with particular sensitivity to nanolite detection through analysis of the 200–1200 cm^−^^1^ spectral region. Differential scanning calorimetry (DSC) enabled quantitative assessment of crystallisation onset temperatures under controlled thermal conditions. Raman spectroscopic analysis demonstrated the absence of detectable nanolites in all starting materials (Supplementary Figs. S2 and S3), confirming complete vitrification. To further probe the stability of the amorphous state, we examined sample LPt1 (see Methods), subjected to 1 h at 1200 °C (Δ*T*_sh_ = 88 °C) followed by 30 min at subliquidus conditions (1100 °C) where clinopyroxene crystallisation is thermodynamically favoured. Remarkably, the Raman spectra of LPt1 revealed no evidence of nanolite formation or incipient crystallisation. The glass maintained a fully polymerised structure indistinguishable from reference superheated glasses LP4 and LP17 (see Methods), indicating that superheating effectively suppressed nucleation even under conditions favourable for crystallisation. These findings demonstrate that superheating not only eliminates pre-existing nucleation sites but also fundamentally alters the melt’s propensity for subsequent crystallisation, consistent with enhanced structural homogenisation at elevated temperatures.

**Fig. 3:**
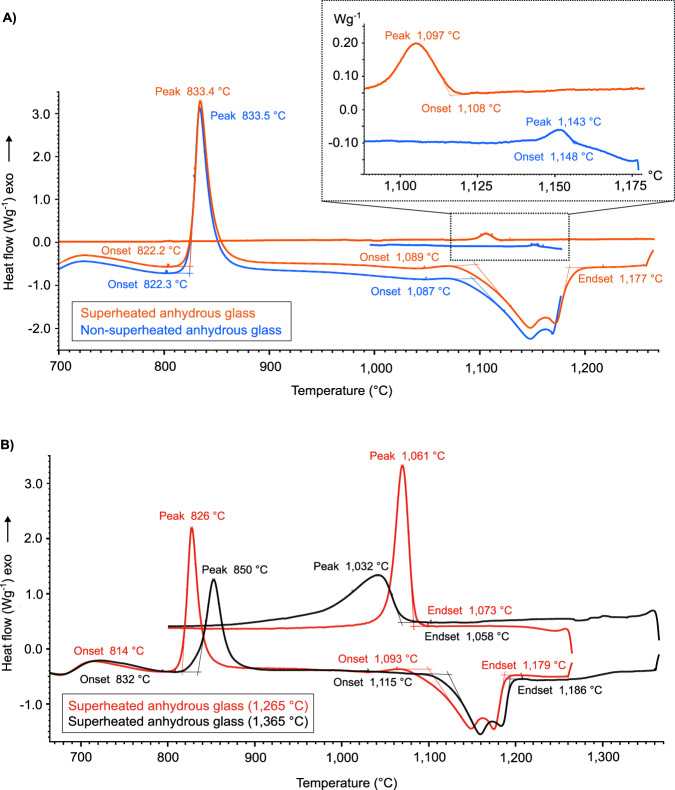
Calorimetry spectra showing crystallisation peaks in glasses. Differential scanning calorimetry (DSC) spectra for (**A**) anhydrous glasses without superheating (blue) and with superheating (orange), and (**B**) glasses subjected to progressively higher degrees of superheating. Data are plotted as differential heat flow (W g^−^^1^) versus temperature (°C).

Differential scanning calorimetry (DSC) was employed to investigate the influence of superheating on the crystallisation behaviour of anhydrous tephritic glass during continuous heating and cooling (Fig. 3; see Methods for details). During continuous heating experiments (Fig. 3a), crystallisation onset occurs at ~820 °C (with a crystallisation peak at ~830 °C) in the anhydrous tephritic glasses heated to liquidus (~1180 °C) and superliquidus (1265 °C) temperatures with a heating ramp of 1 °C min^−1^. These early crystallisation events during heating indicate that nanolites begin to nucleate during the heating ramp, well before liquidus conditions are reached, highlighting the crucial role of heating in initiating nanolite formation. In experiments where the melt was first heated to 1265 °C, well above the clinopyroxene liquidus (*T*_Lcpx_ = 1180 °C), and cooled at a rate of 1 °C min^−1^, crystallisation onset was recorded at 1108 °C, with a crystallisation peak at 1097 °C (Fig. 3a; orange line). In contrast, non-superheated melts, cooled directly from the liquidus, exhibited significantly earlier crystallisation, with onset at 1148 °C and a peak at 1143 °C (Fig. 3a; blue line). To further quantify the relationship between superheating and crystallisation kinetics, additional DSC experiments were conducted using progressively higher superheating temperatures, followed by rapid cooling at 20 °C min^−1^ (Fig. 3b). Melts superheated to 1265 °C exhibited delayed crystallisation onset at 1073 °C (peak at 1061 °C), while those heated to 1365 °C showed an even greater delay, with onset at 1058 °C and peak at 1032 °C. These results clearly demonstrate that increasing the degree of superheating systematically suppresses the nucleation of clinopyroxene, requiring larger undercooling to trigger crystallisation. Combined observations from DSC and in situ view experiments conducted near the liquidus suggest that incipient crystalline clusters or nanolites, possibly formed during the initial heating ramp, may act as heterogeneous nucleation sites and significantly reduce incubation times. Conversely, superheating leads to the dissolution of such structures, yielding a more structurally homogeneous and disordered melt. This homogenisation increases the thermodynamic and kinetic barriers to nucleation, thereby extending the nucleation delay by requiring greater undercooling to re-establish favourable conditions for crystal formation.

### *Influence of superheating on crystal nucleation and growth rates in mafic magmas*

Crystal nucleation behaviour, and consequently the resulting textures in experimental products, is highly sensitive to the thermal path experienced prior to crystallisation^24,40,66–69^. In natural magmatic systems, superheated magma may arise directly from the mantle and enter in subliquidus conditions within the crust, or from decompression of water undersaturated magmas during ascent, which effectively increases the temperature relative to the liquidus without requiring external heat input^18,20,70^. When magma experiences low and moderate superheating, pre-existing nuclei may persist if the temperature is near or just above the liquidus, as dissolution is limited by sluggish resorption kinetics and restricted elemental diffusivity. These surviving nuclei can then promote heterogeneous nucleation upon subsequent cooling. In contrast, if magma is held at larger superliquidus temperatures for sufficient durations, enhanced elemental diffusivity promotes more efficient dissolution of pre-existing nuclei, suppressing heterogeneous nucleation and favouring crystallisation via homogeneous nucleation during cooling. As a result, crystallisation proceeds via homogeneous nucleation at subliquidus conditions, producing fewer but larger crystals (>100 μm). We observed this difference in our in situ and ex situ view experimental samples (Supplementary Text S1). In the experiments with in situ view, crystals are more numerous and smaller, with a maximum length of ~200 μm, while in the ex situ view experiments, crystals are fewer and larger, with a maximum length of ~400 μm (see Supplementary Text S1). As observed in the literature, the occurrence of pre-existing nuclei strongly affects the crystallisation kinetics (i.e., nucleation and growth)^22,69,71–74^. Thus, to corroborate the observation of superheating influence on crystallisation kinetics of pyroxene crystals in our mafic melts, we measured nucleation as the number of crystals per area (Supplementary Data 2 and Methods Section) and the apparent time averaged growth rate (Supplementary Data 3). This latter was calculated following Arzilli et al.^75^ and Bonechi et al.^76^:

1 $${Gr}=\frac{{\left(L * W\right)}^{0.5}}{\left(t * 2\right)}$$

where *L* is the length and *W* is the width of the crystals, and *t* is time.

**Fig. 4:**
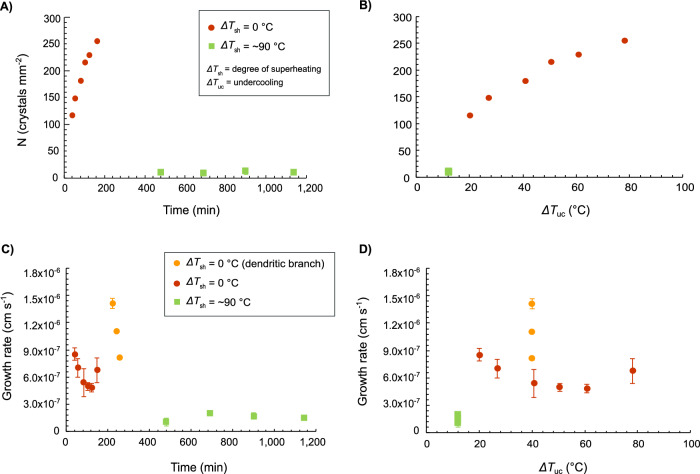
Nucleation and growth rate as function of time and undercooling. Diagrams showing nucleation (number of crystals per area) as a function of (**A**) time and (**B**) undercooling (Δ*T*_uc_), and crystal growth rate as a function of (**C**) time and (**D**) undercooling for in situ (LP1-CC05; Δ*T*_sh_ = 0 °C) and ex situ (Δ*T*_sh_ = ~90 °C) view experiments. Error bars represent standard deviation calculated from measurements of multiple crystals within each experiment.

Regarding nucleation, the in situ view experiments show a progressive increase in the number of crystals per unit area over time (Fig. 4a), consistent with the occurrence of heterogeneous nucleation, as also observed by Polacci et al.^36^. This increase is further enhanced by the continuous increase in Δ*T*_uc_ during the cooling ramp (Fig. 4b, Supplementary Data 2): as Δ*T*_uc_ increases, the thermodynamic driving force for nucleation rises, promoting the formation of additional crystals over time. In contrast, the ex situ view experiments exhibit a nearly constant and lower number of crystals per unit area, suggesting limited or delayed nucleation under those conditions (Fig. 4a). This behaviour is analogous to the multi-step decompression experiments described by Hammer^40^, where slower decompression and smaller instantaneous undercooling led to reduced nucleation efficiency and delayed attainment of chemical equilibrium. Similarly, in our ex situ view experiments, the combination of higher pressure and nearly constant undercooling appears to limit nucleation rates, consistent with Hammer’s observations.

These experimental differences can be understood in terms of the combined effects of undercooling and the melt’s thermal history. Undercooling remains the primary thermodynamic driving force for crystallisation, controlling nucleation and growth and strongly influencing crystal number density, nucleation and growth rates once crystallisation begins. Greater undercooling generally promotes higher nucleation rates and faster crystal growth. However, at comparable degrees of undercooling, prior superheating can significantly affect crystallisation kinetics^24^. Extensive superheating can modify the melt structure, for example, by dissolving pre-existing clusters or structural heterogeneities, thereby delaying nucleation upon cooling into subliquidus conditions. As a result, larger superheating may reduce the effective nucleation rate and crystal number density, and alter subsequent growth behaviour. In summary, while undercooling drives crystallisation at subliquidus conditions, the melt’s superheating history exerts a critical kinetic influence. Consequently, the observed crystallisation behaviour reflects the combined effects of undercooling and superheating, explaining the higher number of crystals observed in the in situ experiments compared with ex situ ones.

Crystal growth rates are generally faster in the in situ (1 × 10^−6^ to 5 × 10^−7^ cm s^−1^) than in the ex situ (~2 × 10^−7^ cm s^−1^) view experiments. Particularly, in the in situ view experiments (Fig. 4c), growth rates decrease over time up to ~150 min, after which a faster growth phase is observed (*t* > ~200 min), associated with the development of dendritic branches in clinopyroxene, particularly in experiment LP1-SSC. Faster growth rates of pyroxene in presence of dendritic branches have been previously observed by Arzilli et al.^4^. For the in situ LP1-CC05 experiment, we also observe the influence of undercooling, with growth rates decreasing as undercooling increases from ~20 to ~80 °C (Fig. 4d; Supplementary Data 3). This trend reflects the concurrent increase in nucleation rate at higher undercooling, which promotes the formation of a larger number of crystals and limits the growth of individual grains^69^. Despite the influence of undercooling, both nucleation and growth rates, moreover, show a clear inverse relationship with the degree of superheating (Fig. 4). While undercooling primarily controls the rate of crystal nucleation and growth once crystallisation begins, higher degrees of superheating determine the initial structural state of the melt prior to crystallisation by promoting efficient dissolution of pre-existing nuclei and enhanced melt homogenisation. As a consequence, prolonged dwell times at superliquidus conditions (Δ*T*_sh_ = ~90 °C), as observed in the ex situ view experiments, result in reduced availability of active nucleation sites, leading to suppressed nucleation and lower crystal growth rates even at comparable degrees of undercooling (∆*T* at the onset of nucleation; Supplementary Data 1).

### *Implications for pre-eruptive crystal content in mafic magmas and magma ascent dynamics*

Our in situ and ex situ view experiments demonstrate that superheating exerts a strong control on crystal nucleation and growth kinetics, which in turn modulate the dynamics of magma ascent. The volume fraction of crystals present in the magma prior to eruption has a strong influence on magma rheology, which governs ascent rates, fragmentation efficiency, and outgassing behaviour, key parameters that ultimately determine eruptive style^2,6,77–80^. Notably, studies by Bamber et al.^77^ and La Spina et al.^6^ demonstrate that even modest variations in magma storage conditions, such as crystal content or pre-eruptive temperature, can lead to substantial changes in the style and intensity of basaltic eruptions.

Constraining whether magma stalled at specific thermodynamic conditions prior to eruption is critical for understanding ascent dynamics and resulting eruptive behaviour. If magma ascends directly from the mantle or a deep reservoir, it is likely to experience significant superheating immediately before eruption. Such superheating may arise from adiabatic or isothermal decompression, particularly under water undersaturated conditions^18,20,70^. Under these conditions, magmas largely retain their initial temperatures, and volatiles remain dissolved in the melt, limiting cooling associated with degassing and allowing magmas to ascend under superliquidus (i.e., superheated) conditions. The degree of superheating depends on the magma’s liquidus temperature, which is controlled by its H_2_O content and initial oxygen fugacity^81^. Once magmas reach shallower crustal levels, they may be stored in reservoirs where cooling is more likely, promoting subliquidus conditions. Consequently, superheating is significantly less likely to develop in shallow reservoirs than in rapidly ascending, deeper-sourced magmas.

In low-viscosity magmas such as basalts, superheating can inhibit crystallisation by inducing a long nucleation delay, thereby facilitating rapid ascent and potentially producing lava fountaining behaviour. Conversely, if magma resides at intermediate or shallow depths for sufficient time before eruption, nucleation delay is minimised. This promotes syn-eruptive crystallisation of microlites during ascent, even under rapid decompression conditions, thereby increasing magma viscosity. Arzilli et al.^61^ demonstrated that syn-eruptive crystallisation in fast-ascending basaltic magmas can occur within tens of seconds, rapidly enhancing viscosity and strain rate, potentially leading to explosive Plinian or sub-Plinian eruptions. In contrast, slower magma ascent allows crystallisation to proceed more gradually, increasing viscosity while simultaneously reducing ascent velocity. This promotes efficient outgassing and favours the development of effusive eruptive behaviours.

**Fig. 5:**
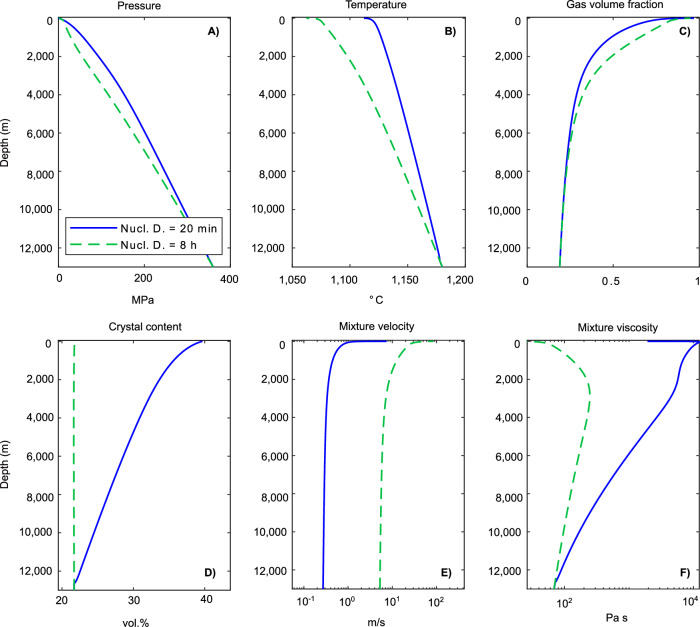
Numerical results for the 2021 Tajogaite eruption reference test case. Numerical solutions computed for high degrees of superheating (large nucleation delay; green lines) and low degrees of superheating (short nucleation delay; blue lines) test cases using the reference input parameters (Supplementary Text S2) and assuming brittle fragmentation criterion. Here we illustrate the profile of (**A**) pressure, (**B**) temperature, (**C**) gas volume fraction, (**D**) crystal content, (**E**) mixture velocity, (**F**) and mixture viscosity.

To evaluate the potential impact of nucleation delay due to superheating on magma ascent dynamics, we conducted numerical simulations using a one-dimensional, steady-state conduit flow model^6,77,82,83^. The model was further developed to explicitly account for nucleation delay (see Supplementary Text S2), allowing us to investigate its influence on magma ascent dynamics^84^. We use the 2021 Tajogaite eruption (La Palma, Spain) as a representative case study. Our aim is not to reproduce the 2021 eruptive activity at La Palma, but rather to illustrate how different nucleation delays may affect the overall magma ascent dynamics and related eruption style in a mafic system. For the numerical simulations we consider two end-member scenarios. In the first scenario, superheated magma rapidly enters the magma reservoir at ~13 km depth, cools to liquidus conditions, and entrains phenocrysts from the resident magma before entering the conduit for the final ascent to the surface. As a consequence of this rapid transit, the residence time in the reservoir is short and therefore negligible compared to the nucleation delay expected under superliquidus conditions. In this case, assuming that the magma was at a high degree of superheating before entering the magma reservoir, we impose a nucleation delay of 8 h, according to our experimental results. In the second scenario, the superheated magma enters, circulates and cools down within the reservoir for a timescale much longer than the nucleation delay expected under superheated conditions. Assuming that the magma chamber temperature is close to liquidus conditions, we impose a nucleation delay of 20 min upon conduit entry, consistent with experimental estimates at near-liquidus conditions. Simulation results are shown in Fig. 5 and Supplementary Fig. S4. Initial and boundary conditions used in the model are detailed in the Supplementary Text S2.

The numerical simulations with a large nucleation delay (i.e., 8 h) due to a high degree of superheating, show that magma ascends from a depth of 13 km^49,82^ without significant crystallisation, transporting to the surface only the crystal cargo inherited from depth (~20 vol.%^82^). The total ascent time in this case is approximately 30 min, which is much shorter than the expected nucleation delay under high degrees of superheated conditions, supporting the absence of syn-eruptive crystallisation. This scenario yields a high mixture velocity and mass eruption rate (~4 × 10^4 ^kg s^−1^), consistent with lava fountaining behaviours. Assuming a nucleation delay of 20 min, reflecting negligible nucleation delay due to a long residence in the magma reservoir at near-liquidus conditions, magma ascends more slowly, over approximately 11.5 h, allowing crystallisation of ~20 vol.% microlites during ascent. This results in a total crystal content of ~40 vol.% at the vent. The increased crystallinity reduces ascent velocity and promotes outgassing, yielding conditions compatible with an effusive eruption and a mass eruption rate of ~2 × 10^3 ^kg s^−1^ (~0.7 m^3^ s^−1^). Our simulations demonstrate that nucleation delay linked to superheating can produce substantial differences in crystallinity, mixture velocity, viscosity, and mass discharge rate (Fig. 5). When placed in the context of the 2021 Tajogaite eruption, these modelled end-member mass eruption rates span a range comparable to the observed variability in eruptive output. Reported values for Tajogaite indicate mass eruption rates ranging from ~1.4 × 10^3 ^kg s^−^^1^ during waning phases to peaks of ~3 × 10^4^–1.8 × 10^4 ^kg s^−^^1^ during early high-flux activity and Strombolian activity, with short-time-window measurements reaching up to ~3 × 10⁴ kg s^−^^1^ during intense fountaining episodes^60,85,86^. The difference between our two modelled end-members (~2 × 10^3^ to ~4 × 10^4 ^kg s^−^^1^) therefore falls within the same order of magnitude as the natural variability observed at Tajogaite, suggesting that superheating-controlled nucleation delay could plausibly contribute to modulation of eruptive intensity within the broader envelope of eruptive behaviour. It is important to highlight that pre-eruptive thermodynamic conditions primarily control magma ascent dynamics and timescale^87,88^. However, when the timescale of ascent is comparable with the timescale of nucleation delay, the latter becomes a critical factor in governing magma ascent dynamics. Recent petrological studies of the 2021 Tajogaite eruption^50,54,57,58,89^ document temporal variations in magma composition, crystal textures, and melt properties, indicating changes in storage and ascent conditions during the eruption. The coexistence of multiple active vents exhibiting different eruptive behaviours (i.e., ash emissions, lava fountains, Strombolian activity, and sustained lava flows)^59,60,85,86,90^ further suggests spatial heterogeneity in conduit and shallow plumbing conditions, which is consistent with a range of local physical environments affecting magma ascent. Within this framework, superheating-induced delays in crystal nucleation provide a physically plausible mechanism that may modulate magma rheology and ascent efficiency under specific thermal conditions. Rather than explaining eruptive style variations at Tajogaite, our results should be viewed as demonstrating how kinetic controls on crystallisation can contribute to variability in ascent dynamics and eruptive parameters in basaltic systems. More broadly, these findings highlight the importance of incorporating superheating effects into models of conduit processes, as they may significantly affect eruptive style across a range of volcanic systems.

## Methods

### *Starting material*

The natural starting material used for the experiments is a tephrite from Tajogaite cone in the Cumbre Vieja ridge (La Palma, Canary Islands) erupted on 2 October 2021 (CVLP-LF3 sample^60^; Supplementary Data 4).

In situ view experiments. The natural crushed rock was melted at 1400 °C for 4 h in a Pt crucible placed in a 1-atm box furnace at The University of Manchester (United Kingdom). This melting time has allowed the natural powder to fully degas and to dissolve any crystals present. After that time the crucible was removed from the furnace and cooled in air. The melting procedure was repeated twice to enhance homogenisation. The hydrous starting glass (LP1) was obtained by melting the recovered glass and homogenising it with 1 wt.% H_2_O added in Au_80_Pd_20_ capsules at 1250 °C and 60 MPa for 24 h using an internally heated pressure vessel apparatus at the Institute of Earth System Sciences, Leibniz University of Hannover (Germany).

Ex situ view experiments. The hydrous starting material (1 wt.% H_2_O added) used for the ex situ view experiments was prepared by mixing powder of the natural anhydrous tephrite (CVLP-LF3)^60^ with a hydrous-rich (5 wt.% H_2_O added)^49^ synthetic analogue in the ratio 80:20.

### *In situ view synchrotron X-ray microtomography experiments*

Intermediate pressure, high temperature (IPHT) experiments with in situ view were performed at the X-ray tomography beamline I12-JEEP, Diamond Light Source, Harwell, United Kingdom. We used a dedicated X-ray transparent Internally Heated Pressure Vessel (IHPV^62^) apparatus developed at Neel Institute (France) and based on a previous one^91,92^ combined with X-ray microtomography to perform crystallisation experiments with in situ view under water saturated conditions at crustal pressures. With respect to the original setup used in Bonechi et al.^62^, the bottom part of the IHPV has been modified to enable X-ray microtomography acquisition (Supplementary Fig. S5). This adaptation includes a customised connection to a rotation mechanism, allowing the sample holder to rotate independently within the vessel. The rotation system consists of a motor which is magnetically coupled to the modified base of the vessel via paired magnets. This configuration enables sample rotation within the IHPV, allowing full tomographic acquisition through the fixed sapphire windows without requiring the entire vessel to rotate. The pressurisation was controlled by a pressure regulator^93^. The IHPV is characterised by the placement of the furnace inside the vessel (internally heated). The vessel is a thick-walled steel cylinder having both ends open. The open ends are closed by heads through which pressure, electrical and thermocouple lead enter. Pressure and temperature are confined respectively by two external and two internal sapphire windows at 180°, which allow the X-ray beam to enter the vessel, passing through the sample, and reaching the camera for microtomography acquisitions. Temperature was measured with a K-type thermocouple positioned close to the sample in the middle of the furnace hot spot. The K-type thermocouple measures the sample temperature with an uncertainty of ±0.5 °C. The sample holder was an alumina, which is suitable for the temperature range investigated and has a low X-ray attenuation coefficient. The hydrous glasses were placed in the cylindrical alumina crucible (outer diameter 5 mm, and inner diameter 4 mm). We pressurised the system at first with gas (He) up to 20 MPa, and then we heated up to 1140 °C. We kept then the system at 1140 °C for 20 min. At this point we continued the experiments via (1) single step cooling (SSC; Δ*T* = ~20 °C) at temperatures ranging between 1140 and 1080 °C with a cooling rate of 45 °C min^−1^ between each step (LP1-SSC), and (2) continuous cooling (CC) at cooling rates of 0.5 °C min^−1^ (LP1-CC05) and 1 °C min^−1^ (LP1-CC1). We worked at fixed pressures typical of shallow to intermediate crustal storage (*P* = 20–50 MPa, corresponding to a depth of ~1–2 km), under H_2_O-saturated conditions (Supplementary Fig. S6).

### *Ex situ view experiments*

The experiments were carried out at the Experimental Petrology Laboratory of the Institute of Petrology and Structural Geology (Charles University, Prague, Czech Republic) in a Quick press non-end loaded piston cylinder apparatus at 275 MPa in the temperature range 1100–1200 °C. The starting mixture (about 20 mg) was loaded inside cylindrical capsules (Au_80_Pd_20_, OD: 3 mm, ID: 2.7 mm), that were sealed by arc-welding under a flux of argon. The employed 19–25 mm NaCl-pyrex-graphite-MgO low-pressure assemblies impose relatively oxidising conditions (NNO + 2)^94^. S-type (Pt-Pt_90_Rh_10_) thermocouples were used to measure the temperature with an accuracy of ±2 °C. The thermal history of each experiment consist of heating it at a rate of 15 °C min^−1^ to 700 °C, with a rate of 50 °C min^−1^ to 1200 °C (superliquidus), maintaining the superliquidus temperature for 1 h, cooling it at a rate of 1 °C min^−1^ to 1100 °C, holding it at 1100 °C for variable time from 0.5 to 19 h (Fig. 1). All experiments terminated with isobaric quench by switching off the power (cooling rates >100 °C s^−1^).

### *In situ synchrotron X-ray microtomography acquisition*

The X-ray tomography beamline I12-JEEP (Diamond Light Source, Harwell, United Kingdom) allowed us to perform experiments in phase-contrast mode, setting the sample-to-detector distance at 2200 mm in order to work in the edge-detection regime^95^. The tomographic projections were acquired using a monochromatic X-ray beam with energy of 53 keV. In each scan, 1800 tomographic projections were acquired by the detector with equiangular steps (2.174 deg s^−1^) over a full rotation angle of 180°. The exposure time for the acquisition of each projection was 0.046 s, thus the temporal resolution of each scan is ~83 s. The isotropic pixel size was 3.24 μm. The detector is a high-resolution imaging pco.edge 5.5 camera with optical module 3, corresponding to a field of view of 8.0 × 7.0 mm. Scan acquisition started during the heating ramp and covered the entire duration of the experiment.

### *Image reconstruction*

Tomographic projections were reconstructed into 2D slices by using Diamond I12 in-house python codes, using the Gridrec algorithm, implemented in Savu plugin (https://savu.readthedocs.io/en/latest/tutorials/confluence/I12/SAVU-Tomography-Reconstruction.html). The pre-processing pipeline includes centre of rotation calculation^96^, zinger removal, blob removal^97^ and regularisation-based ring removal^98^. The reconstructed slices were converted to 8-bit raw format and stacked using ImageJ software (v. 1.54 d)^99^ to obtain volumes in which the isotropic voxel has an edge size of 3.24 μm.

### *Image processing and segmentation*

Segmentation is the process that allows separation of objects from the background to obtain binary volumes containing only the feature of interest. Segmentation of pyroxene crystals from the glassy matrix was performed using Avizo® software. Before segmentation a 3D Non-Local Mean filter in Avizo® software was applied to smooth the greyscale input images; this allows us to better distinguish and segment pyroxene crystals from their glassy matrix, reducing the noise and the potential artefacts whilst preserving edges and the shapes of the objects. Segmentation of pyroxene crystals from the glassy matrix was operated in the 3D domain with Avizo® software by using manual bi-level greyscale thresholding based on the greyscale histogram of the selected VOIs and visual inspection of the slices in different directions (axial, coronal and sagittal).

### *Scanning electron microscope analysis*

BSE images were collected using a JEOL JSM-6390LA FE-SEM in the Department of Earth and Environmental Sciences, The University of Manchester, United Kingdom, to analyse vesicles shapes and crystals morphologies. We used an acceleration voltage of 15 kV and a working distance of 10 mm.

### *Raman spectroscopy*

Raman spectroscopy was conducted on LP1 (1250 °C, 60 MPa, 1 wt.% H_2_O) natural glass used for the in situ view experiments, and on LPt1 (Supplementary Data 1) ex situ superheated glasses to verify the presence of nanolites/nanocrystals. Two additional ex situ superheated glasses, LP4 (1200 °C, 275 MPa, 1 wt.% H_2_O, 24 h) and LP17 (1175 °C, 275 MPa, 1 wt.% H_2_O, 24 h), produced in the work of Fabbrizio et al.^49^ using the same starting material, were also analysed.

Raman spectra were acquired using an Alpha300R WITec Raman microscope at GLASS (Gateway Laboratory of Amorphous and Structured Solids and Melts, CNR–ISSMC). For each sample, 10 spectra were acquired to investigate the experimental reproducibility of the results. The instruments are equipped with a 457 nm blue solid-state laser and a microscope. The laser power on the sample surface was 11 mW through a 100× objective and ~1 μm^2^ spot size. Calibration was performed using a silicon standard. Instrumental settings consisted of 1800 grooves/mm grating density with an exposure time of 10 s and 10 accumulations. The Raman scattering was collected on a polished sample surface over a range from 100 to 1400 cm^−1^. Raman signal was found to be maximised at 6 μm of depth using a motor on the Z axis. Therefore, spectra were collected at the same depth for all samples. Prior to the Raman spectra acquisition, the samples were stored at 100 °C in an oven to avoid water absorption on the surface.

### *Raman spectra treatment*

All the Raman spectra of this work were processed using the Matlab code developed by Di Genova et al.^12^. Following the protocol of Di Genova et al.^12^, Raman spectra intensity were corrected for the frequency-dependence scattering intensity and temperature^100,101^ as following:

2 $$I={{{\rm{I}}}}_{{{\rm{obs}}}}\cdot \{{v}_{0}^{3}\cdot v\frac{[1-\exp (-{hcv}/{kT})]}{{({{{\rm{v}}}}_{0}-{{{\rm{v}}}})}^{4}}\}$$

where I_obs_ is the Raman spectra intensity, *ν*_0_ is the wavenumber of the incident laser light (10^7^/532 cm^−1^ for the green laser), ν is the measured wavenumber in cm^−1^, *h* is the Planck constant (6.62607 × 10^−34 ^J s), *c* is the speed of light (2.9979 × 10^10 ^cm s^−1^), *k* is the Boltzmann constant (1.38065 × 10^−23 ^J K^−1^) and *T* is the absolute temperature. The baseline subtraction procedure is based on a single cubic spline fitting independent on the chemical composition with wavenumber interval for anchor points from ~100 to ~1500 cm^−1^ for silicate region^12^.

### *Differential Scanning Calorimetry*

Thermal analysis was conducted using a Mettler Toledo STA/TGA-DSC instrument at the GLASS Laboratory (CNR–ISSMC) to measure heat flow as a function of temperature. Experiments were performed in Pt_80_Rh_20_ crucibles under nitrogen atmospheres. Approximately 40 mg of the anhydrous tephritic glass was used for each measurement. Instrument calibration for temperature and enthalpy was carried out using the known melting points and enthalpies of fusion of high-purity reference standards (In, Sn, Zn, Al, Au). Baseline corrections were applied using measurements from two empty platinum crucibles.

### *Crystal number per unit area measurements*

The number of crystals per unit area (Supplementary Data 2) was determined using different imaging techniques for in situ and ex situ view experiments. For the in situ view experiments, crystal counts were performed on reconstructed synchrotron X-ray microtomographic slices acquired during the experiments. Crystals were identified and counted directly on the tomographic images over a fixed analysed area. Tomographic slices were used because they provide time-resolved information during crystallisation, allowing quantification of crystals at different stages of growth. For the tomographic datasets, the minimum detectable crystal size is determined by the spatial resolution and the phase-contrast imaging mode, which enables the observation of crystals with dimensions close to the voxel size. Based on these constraints, the smallest crystals that can be reliably detected and measured are approximately 13 µm in length.

Although crystals smaller than 13 µm may be visually identifiable, background noise associated with in situ acquisition prevents reliable segmentation and quantitative analysis. Therefore, crystals below 13 µm were excluded from number density calculations.

For the ex situ view experiments, the number of crystals per unit area was measured on backscattered electron (BSE) images of polished sections. Ex situ measurements necessarily rely on BSE images of the final, post-experiment samples, as these experiments do not capture crystal growth in real time. In both cases, crystal counts were performed using ImageJ (version 1.54d)^99^. The same identification criteria were consistently applied to all datasets to ensure comparability between in situ and ex situ view experiments.

## Supplementary information

Supplementary Information

Description of Additional Supplementary Files

Supplementary Data 1–4

Transparent Peer Review file

## Data Availability

The data supporting the findings of this study are available within the article and its Supplementary Information. Input files required to reproduce the numerical simulations are provided in the Supplementary Information.

## Code availability

The MAMMA code (Magma Ascent Mathematical Modelling and Analysis), developed in Fortran 90 and used in this study to perform numerical simulations, is publicly available on Zenodo at 10.5281/zenodo.19881462.
