# Supplementary material for: Superheating in mafic magmas controls clinopyroxene nucleation delay and magma ascent dynamics
Source: Nat Commun. 2026 Jun 8;17:4962. doi: 10.1038/s41467-026-73352-1 (PMC13247257; doi:10.1038/s41467-026-73352-1)
Supplement: Supplementary file 1 — Supplementary Information [file 41467_2026_73352_MOESM1_ESM.pdf]

Supplementary Information for

Superheating in mafic magmas controls clinopyroxene nucleation delay and magma ascent dynamics

Barbara Bonechi[1*], Fabio Arzilli[2], Margherita Polacci[1], Alessandro Fabbrizio[3], Giuseppe La Spina[4], Eleni Michailidou[5], Elisa Biagioli[1], Richard A. Brooker[6], Jean-Louis Hazemann[7], Robert C. Atwood[8], Danilo Di Genova[9], Sumith Abeykoon[2], David Neave[1], Renat R. Almev[10], Mike Burton[1]

Corresponding author: barbara.bonechi@manchester.ac.uk

1: Department of Earth and Environmental Sciences, The University of Manchester, Manchester, United Kingdom

2: School of Science and Technology, Geology Division, University of Camerino, Camerino, Italy

3: Dipartimento di Scienze dell'Ambiente e della Terra - DISAT, Università degli Studi di Milano-Bicocca, Piazza dell'Ateneo Nuovo, 1-20126, Milano, Italy

4: Istituto Nazionale di Geofisica e Vulcanologia (INGV), Sezione Osservatorio Etneo, Catania, Italy

5: Institute of Petrology and Structural Geology, Faculty of Science, Charles University, Prague, Czech Republic

6: School of Earth Sciences, University of Bristol, Bristol, United Kingdom

7: Université Grenoble Alpes, CNRS, Grenoble INP, Institut Néel, Grenoble, France

8: Diamond Light Source, Harwell Science and Innovation Campus, Harwell, United Kingdom

9: Institute of Science, Technology and Sustainability for Ceramics (ISSMC), National Research Council of Italy (CNR), Faenza, Italy

10: Institute of Earth System Sciences, Section of Mineralogy, Leibniz University Hannover, Hannover, Germany

This PDF contains:

Supplementary Figures S1–S6

Supplementary Text S1–S2

References

Supplementary Figures S1-S6

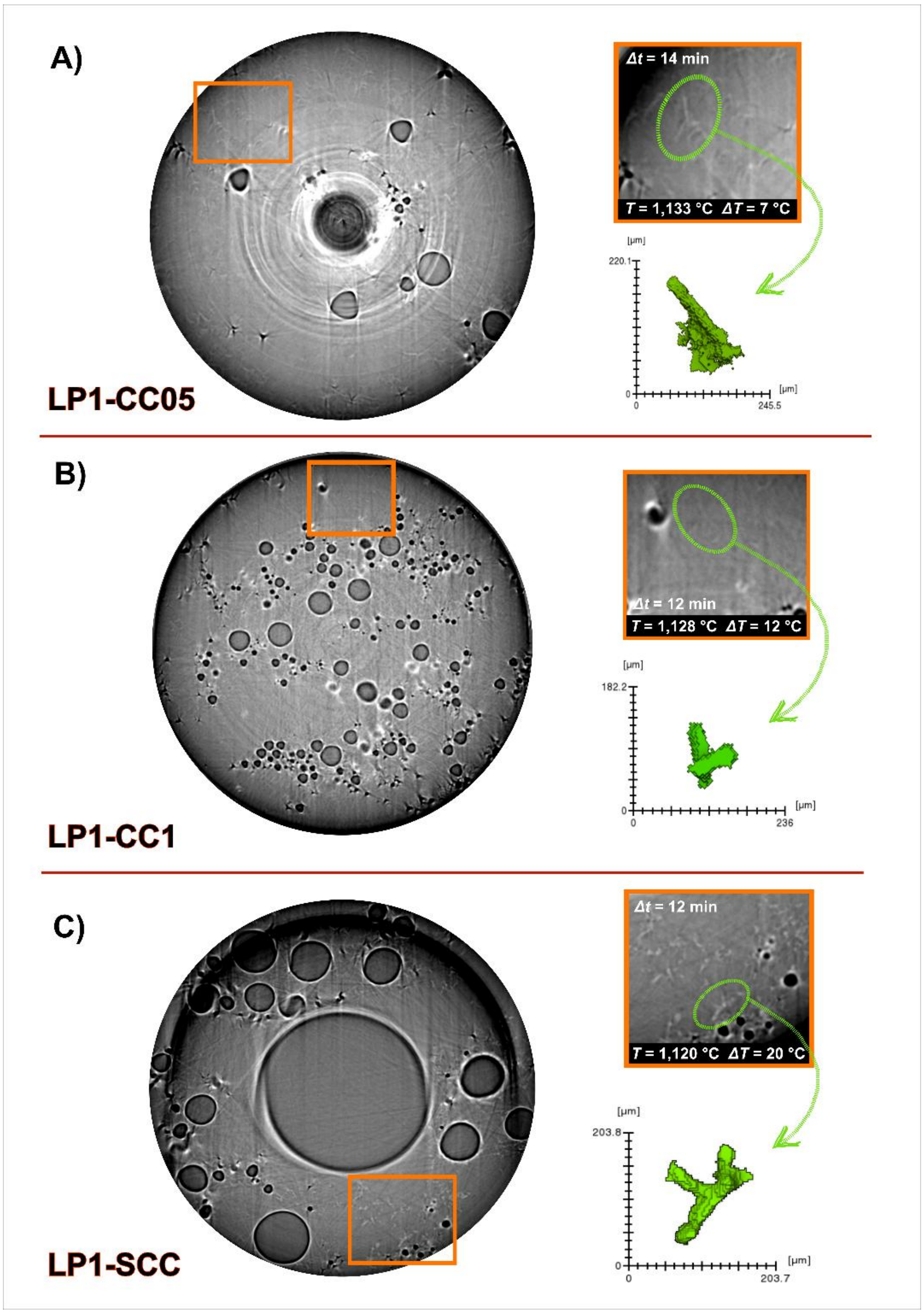

Figure S1. Reconstructed slices and volume renderings of clinopyroxene crystals in *in situ* view experiments. Reconstructed slices showing the appearance of clinopyroxene crystals in A) LP1-CC05, B) LP1-CC1, and C) LP1-SSC experiments with *in situ* view. The insets on the right side of the slices illustrate the volume renderings of the segmented clinopyroxene crystals obtained with Avizo software.

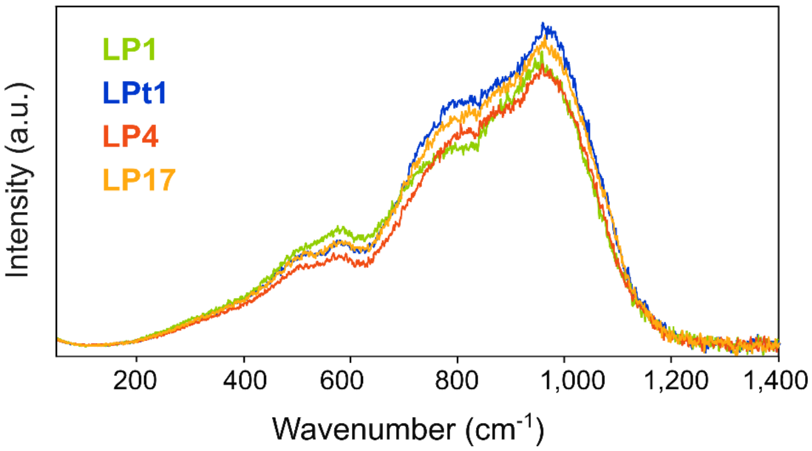

Figure S2. Raman spectra of nanolite-free glasses. Raman spectra (LW region) of nanolite-free natural (LP1) and *ex situ* superheated (LP4, LP17, LPt1) glasses corrected for the excitation line and temperature (Eq. 2) and normalised to the total area.

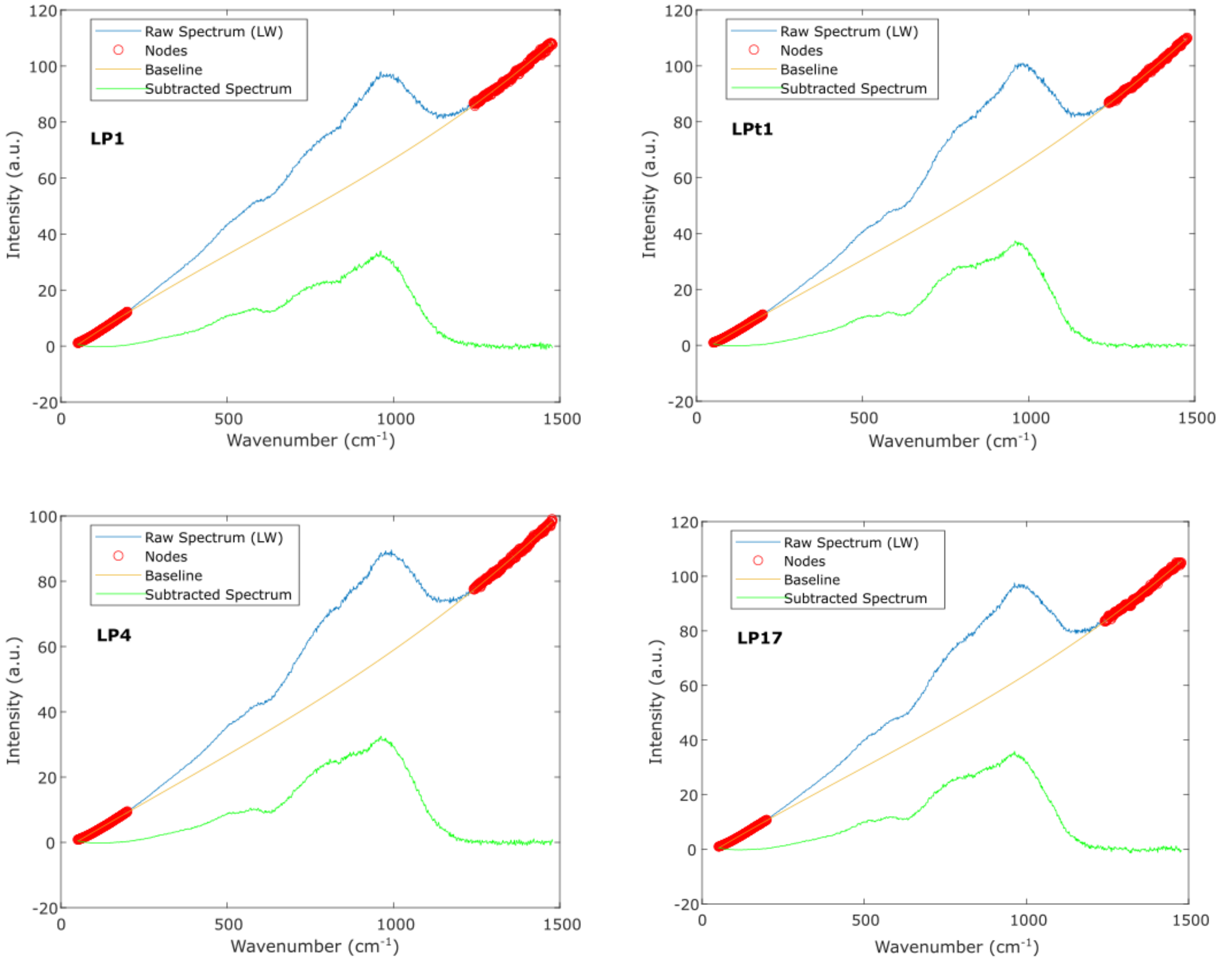

Figure S3. Corrected Raman spectra of LP1 natural glass, and LPt1, LP4 and LP17 superheated glasses for silicate (SR; 100–1,500 cm^{-1}) region. Yellow lines represent the baseline according to the procedure reported in this study.

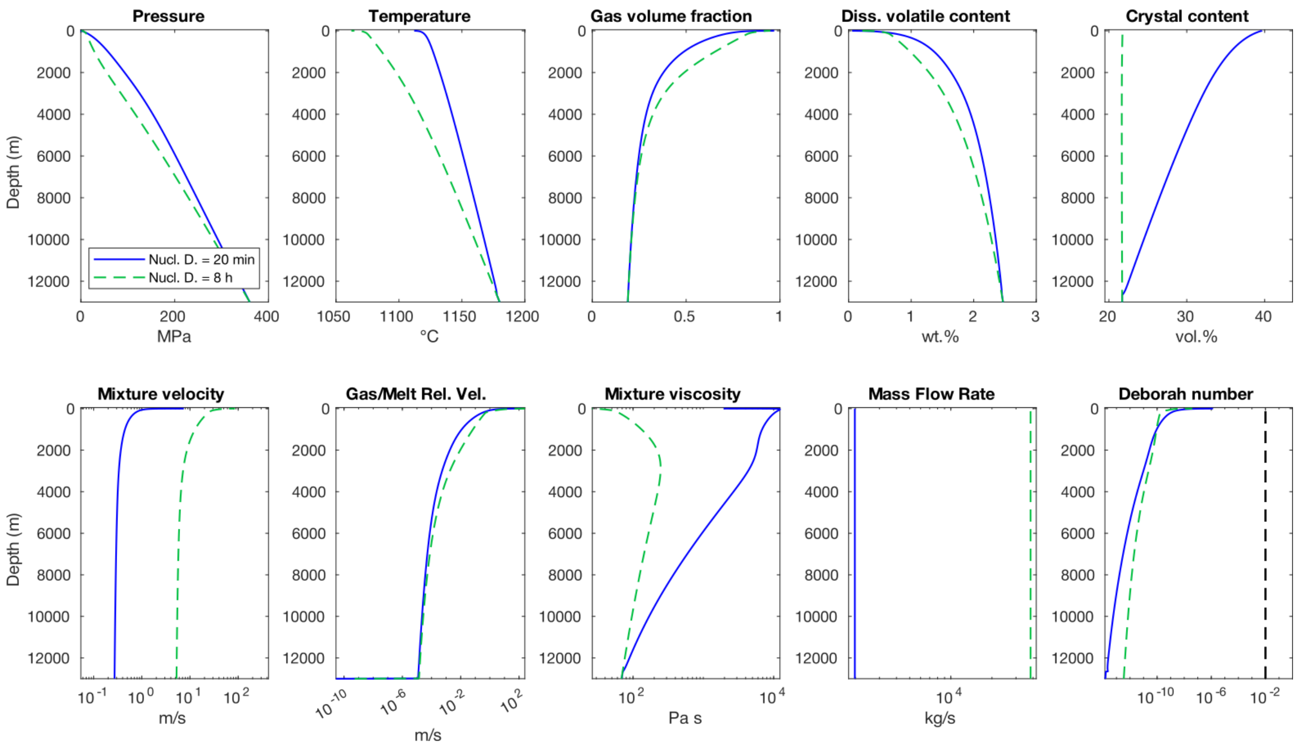

Figure S4. Numerical results for the 2021 Tajogaite eruption reference test case. Numerical solutions computed for high degree of superheating (green lines) and no superheating (blue lines) test cases using the reference input parameters and assuming brittle fragmentation criterion. Black dashed line is the threshold for the fragmentation criterion.

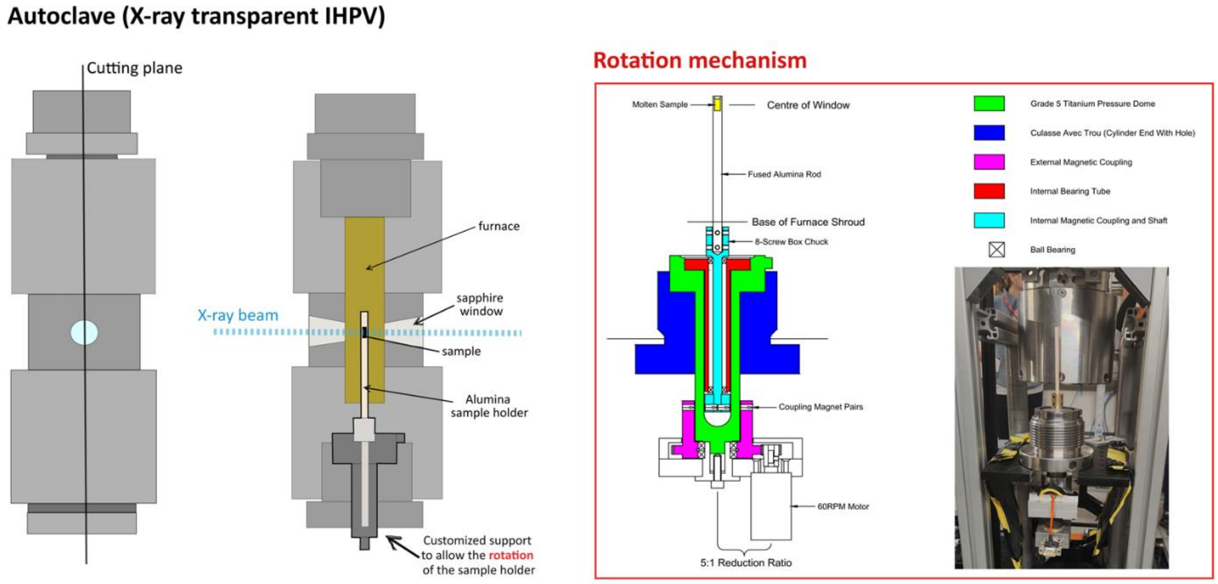

Figure S5. Sketch of the IHPV shown from both external (left) and internal (right) views. The right panel highlights the rotation mechanism specifically developed to enable X-ray tomography acquisition. Adapted from Bonechi et al.[1].

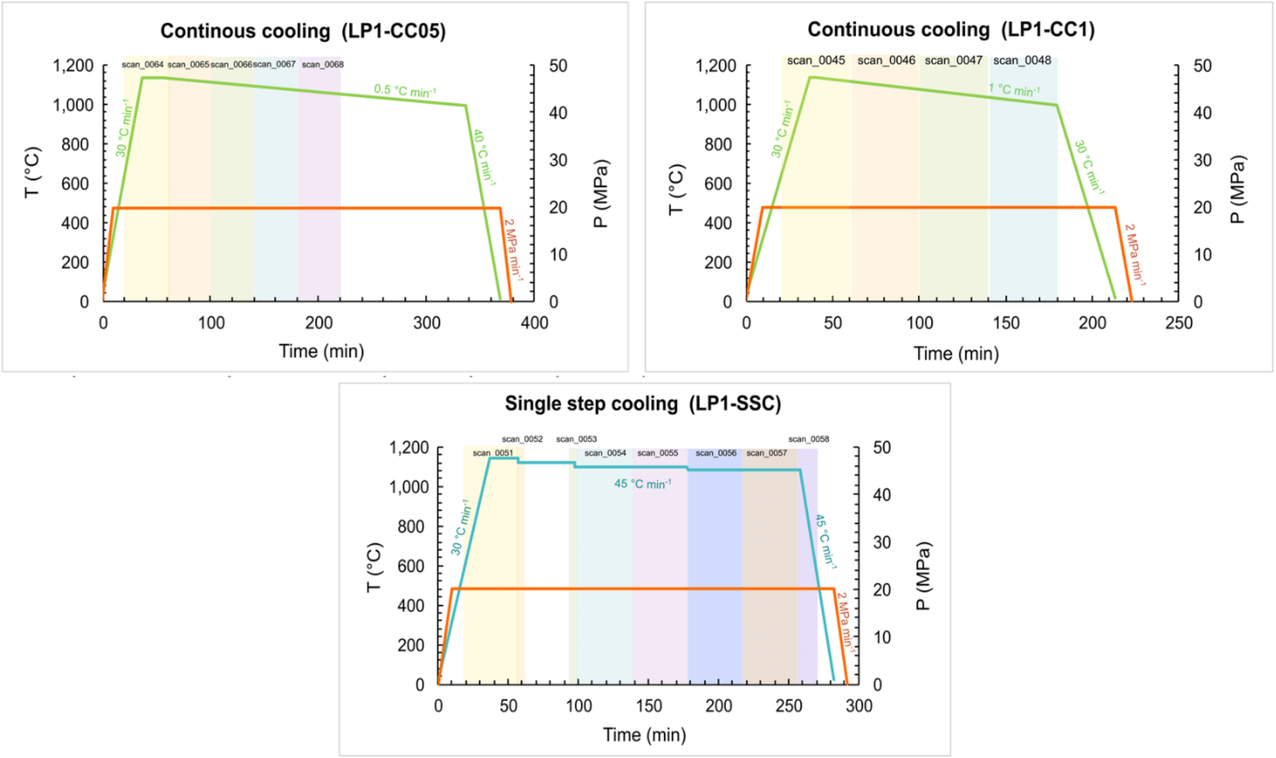

Figure S6. Diagrams show temperature-pressure-time conditions kept during continuous cooling (CC) and single step cooling (SSC) *in situ* view experiments.

Supplementary Text S1-S2

Text S1. Observation from recovered samples

In all *in situ* view experiments, the phase assemblage consists of clinopyroxene, plagioclase, olivine, oxide and glass. Mineral phases crystallised in the continuous cooling experiments show mainly euhedral habit (Figs. S7-S8), while those in the single step experiment are predominantly anhedral (Fig. S9). Euhedral and subhedral clinopyroxene crystals are commonly zoned with hourglass zonation, reaching sizes up to 200 µm (Fig. S7). Dendritic ones, instead, do not show zonation and are smaller in size (<10 µm). Plagioclase commonly crystallised at the edges of the crucible as also observed in previous experiments[2–4], and reach sizes up to 200 µm (Figs. S7-S9). Olivine crystals show growth habit with presence of melt inclusions and reach sizes up to 200 µm. Finally, oxides are usually hexagonal and very small in size (<10 µm) and concentrated around bubble edges.

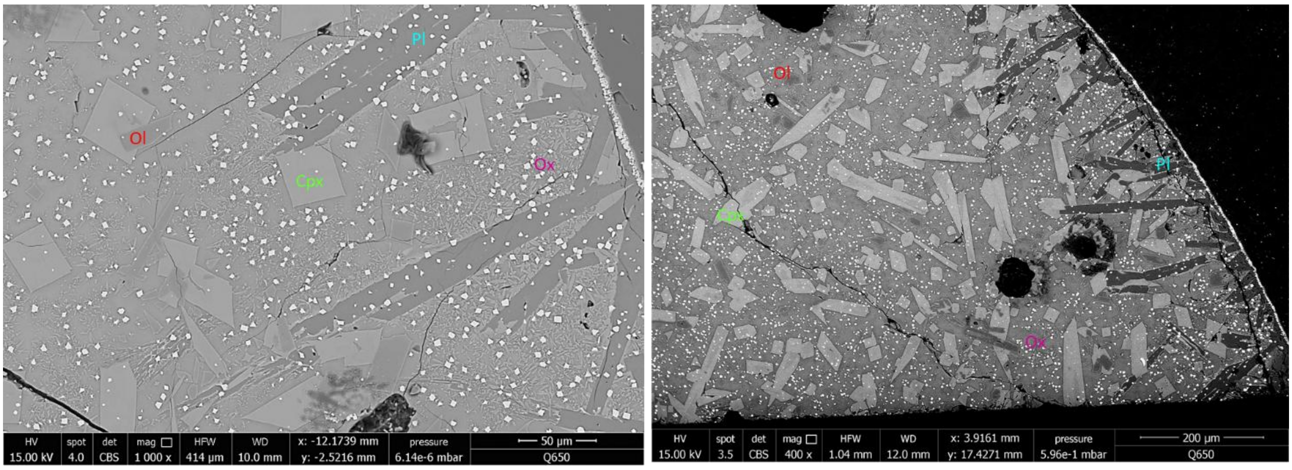

Figure S7. BSE images of the recovered sample LP1-CC05 after the end of the experiment. Cpx = clinopyroxene, Pl = plagioclase, Ol = olivine, Ox = oxide.

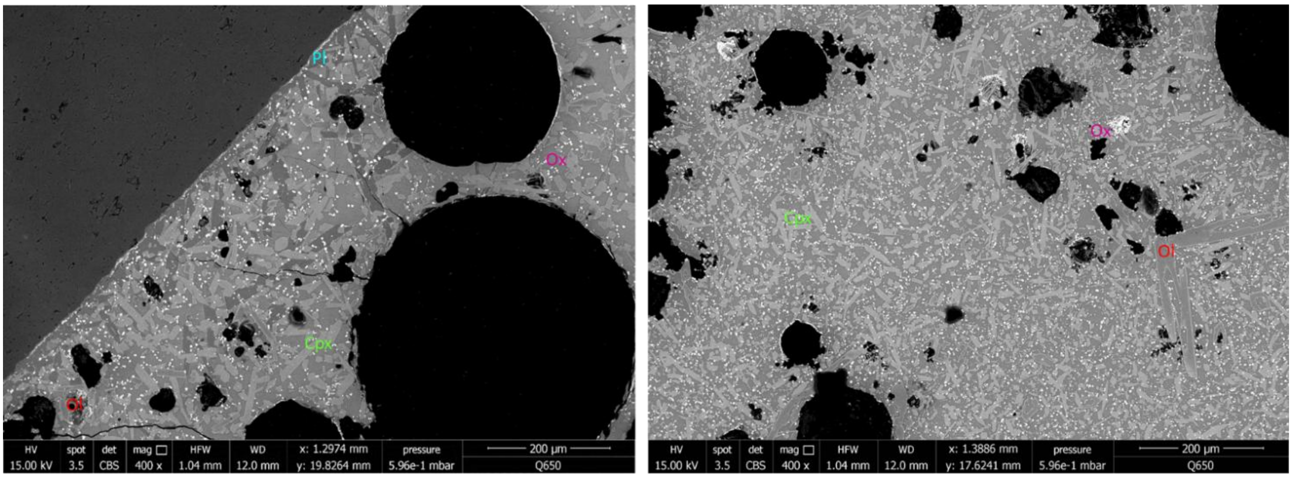

Figure S8. BSE images of the recovered sample LP1-CC1 after the end of the experiment. Cpx = clinopyroxene, Pl = plagioclase, Ol = olivine, Ox = oxide.

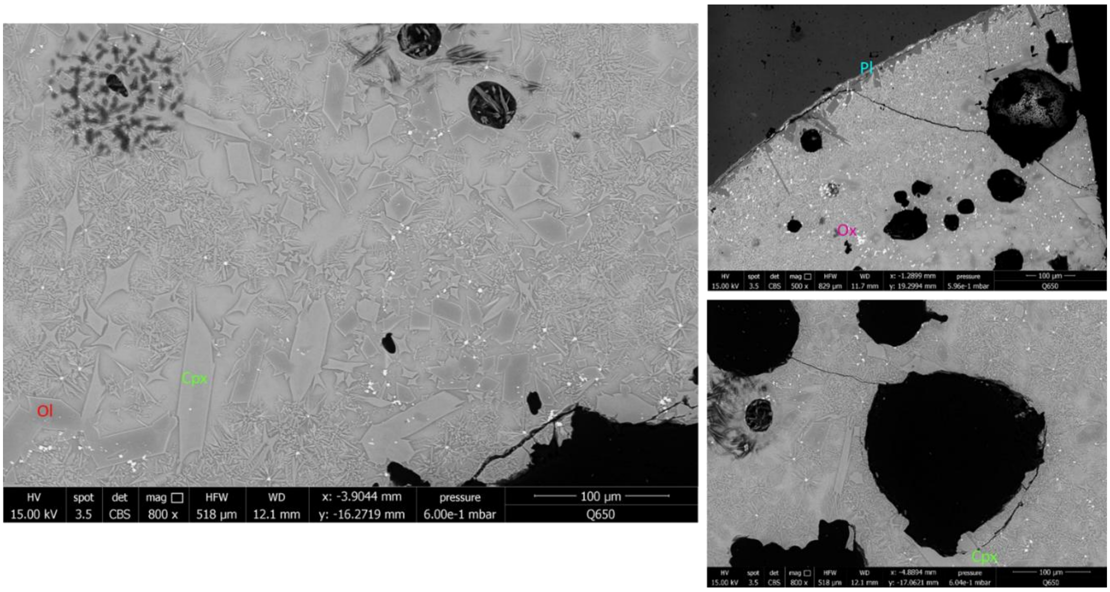

Figure S9. BSE images of the recovered sample LP1-SSC after the end of the experiment. Cpx = clinopyroxene, Pl = plagioclase, Ol = olivine, Ox = oxide.

In the single step cooling experiment (LP1-SSC) there is also the presence of some euhedral clinopyroxene with sizes up to 100 µm and broken in presence of vesicles (Fig. S9). Their presence can be related to the long stay (~3 h) at 1,120 °C, whereas dendritic crystals are probably due to the high cooling rate (45 °C min^{-1}). This is in agreement with the presence of some dendritic crystals also in the continuous cooling experiments, related to the last cooling step. Specifically, there are a few very small dendritic crystals (<5 µm) in the LP1-CC1 (cooling rate = 30 °C min^{-1}), whereas they are more numerous in LP1-CC05 where the final cooling rate was 40 °C min^{-1}. Considering these experiments, we can observe a decrease in the abundance of dendritic crystals with decreasing cooling rate from 45 to 30 °C min^{-1} (Fig. S10).

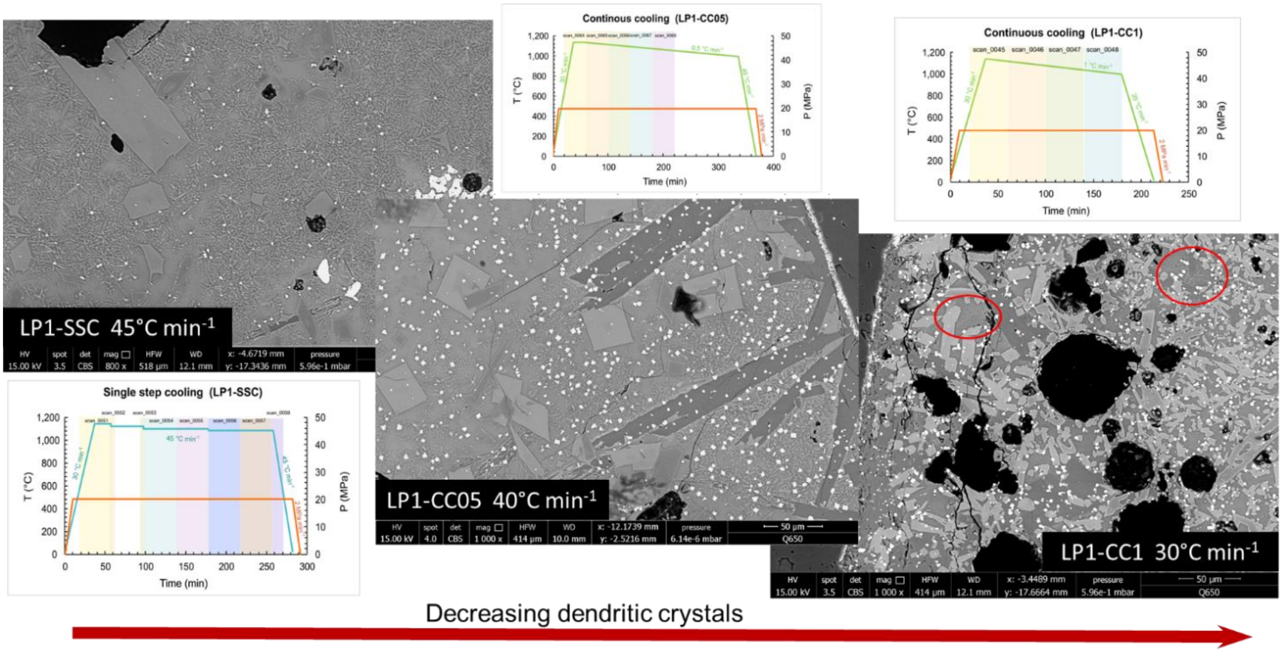

Figure S10. BSE images of the recovered sample LP1-CC05, LP1-CC1 and LP1-SSC after the end of the experiment. Arrow shows the decrease of dendritic crystals as function of cooling rate. Red circle highlights dendritic crystals present in melt pools.

In the *ex situ* view experiments, the mineralogical assemblage change as a function of the resting time at 1,100 °C (Supplementary Data1). Samples remain glassy after 0.5 h; olivine and chromite appear after 1 h, and clinopyroxene crystallises after 8 h. Chromite and zoned clinopyroxene are mostly euhedral, whereas olivine occurs as euhedral crystals hosting melt inclusions or as skeletal shapes with multiple embayments and/or melt inclusions (Fig. S11).
Importantly, clinopyroxene crystals in the *ex situ* view experiments are fewer in number and larger in size, reaching up to ~400 μm (Fig. S11), compared with the smaller (~200 μm; Fig. S7) and more numerous crystals in the *in situ* view experiments (Fig. S12).

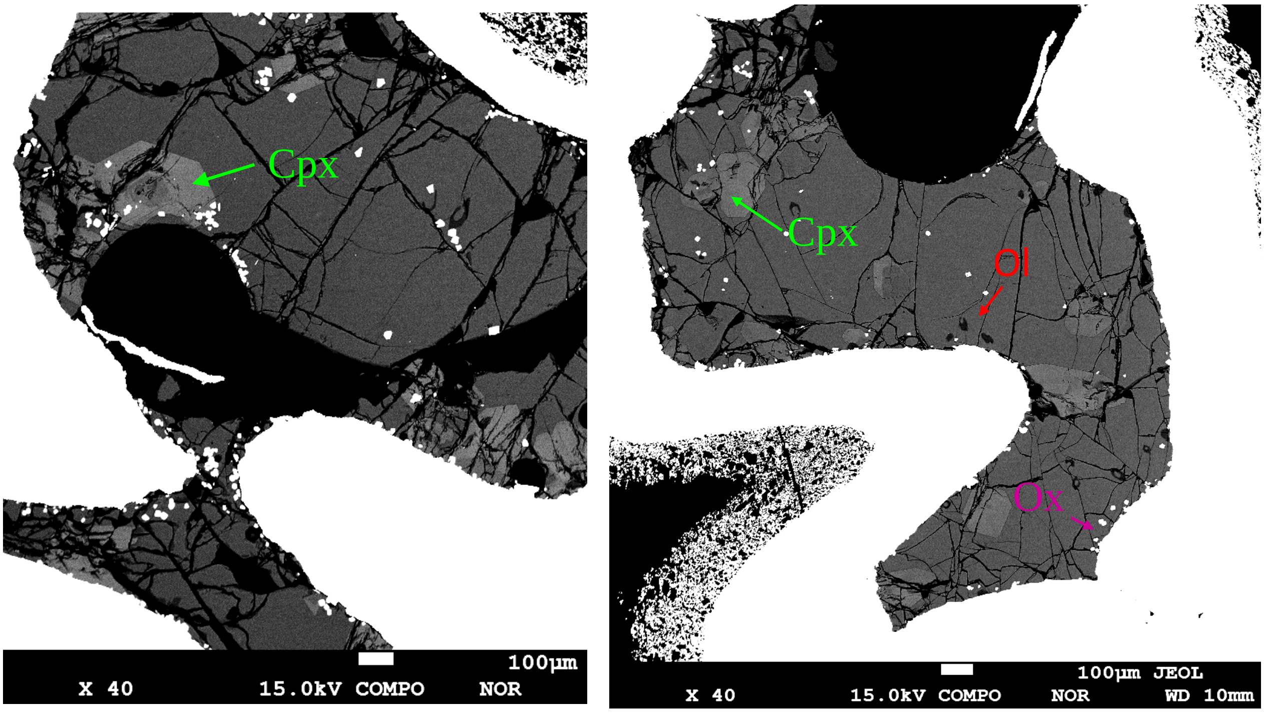

Figure S11. BSE images for ex situ experiments LPt7 (left) and LPt5 (right) showing crystallised phases. Cpx: clinopyroxene; Ol: olivine; Ox: oxide.

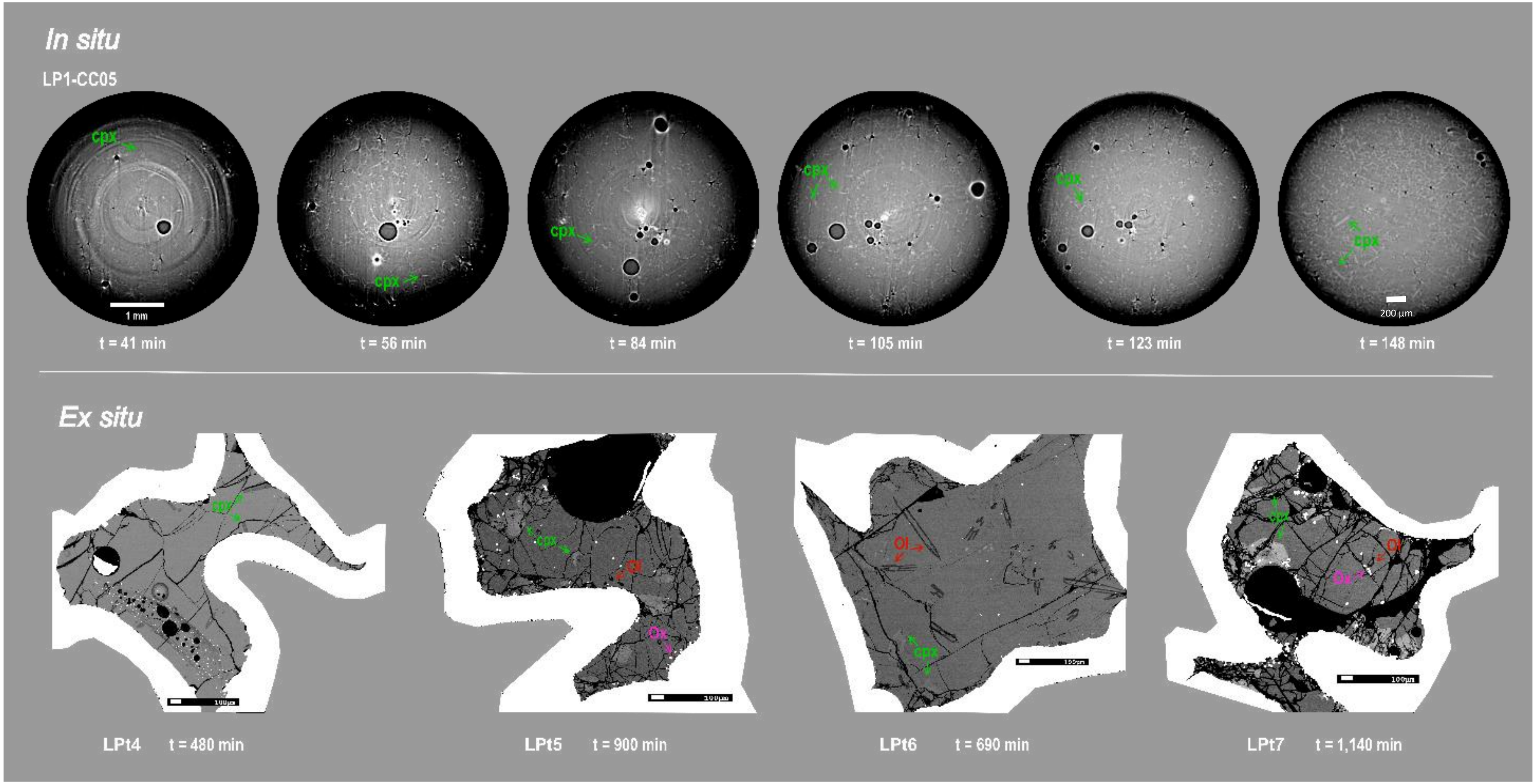

Figure S12. Reconstructed slices for in situ experiment LP1-CC05 and BSE images for ex situ experiments (LPt4, LPt5, LPt6, LPt7) showing clinopyroxene nucleation and growth over time. Cpx: clinopyroxene; Ol: olivine; Ox: oxide.

Text S2. Numerical model and initial conditions for the numerical simulations

To simulate magma ascent dynamics for the 2021 Tajogaite eruption (La Palma, Spain) test case, we further develop the model illustrated in Biagioli et al.[5] to incorporate crystal nucleation delay. To include nucleation delay, the numerical model calculates magma ascent time from magma ascent velocity at each depth. Crystallisation is then neglected until ascent time is equal to the nucleation delay, and only from that depth until the vent of the conduit crystallisation is permitted (following the transport equation for the evolution of the crystal volume fraction). In this framework, the total crystal fraction is derived from extensive Rhyolite-MELTS simulations (over 100,000 runs) that account for all crystallising phases, with the experimentally measured clinopyroxene nucleation delay serving as a representative proxy for the onset of bulk crystallisation in the magma.

Compared to the numerical model adopted by Biagioli et al.[5] we also included the effect of bubbles on magma viscosity, according to Llewellin et al.[6], Mader et al.[7], and La Spina et al.[8].

The rheological model for the melt-crystal free and the relative viscosity due to the presence of crystals are the same as those adopted by Biagioli et al.[5], with the same melt inclusion composition. Similarly, the equilibrium crystallisation model, the characteristic time of crystallisation and exsolution, the friction with the wall of the conduit and the outgassing model are the same as those adopted by Biagioli et al.[5]. As solubility model, instead, we considered the Henry's law for water and CO_2 with solubility parameters from Arzilli et al.[3].

The initial conditions adopted here for simulating the 2021 Tajogaite eruption (La Palma, Spain) are similar to those assumed by Biagioli et al.[5], with some refinement. We considered the same inlet pressure (360 MPa), but a higher inlet temperature of 1180 °C, to have a near-liquidus conditions at the inlet of the conduit. Water and CO_2 contents are 2.0 wt.% and 4.5 wt.%, respectively, in agreement with Biagioli et al.[5]. We assumed a slightly higher initial phenocrysts content (20 vol.%), but still in agreement with textural data from Bonechi et al.[9]. Finally, conduit radius is assumed to be 1 m, in order to obtain a mass flow rate in agreement with in agreement with literature data[10].

References

1. Bonechi, B. et al. The role of superheating in controlling crystallization kinetics: insights from the 2021 Tajogaite eruption. *IAVCEI 2025 Conference Poster* (2025).
2. Arzilli, F. et al. Dendritic crystallization in hydrous basaltic magmas controls magma mobility within the Earth's crust. *Nat. Commun*. **13**, 3354 (2022).
3. Arzilli, F. et al. Magma fragmentation in highly explosive basaltic eruptions induced by rapid crystallization. *Nat. Geosci*. **12**, 1023–1028 (2019).
4. Polacci, M. et al. Crystallisation in basaltic magmas revealed via in situ 4D synchrotron X-ray microtomography. *Sci. Rep*. **8**, 1–13 (2018).
5. Biagioli, E. et al. Numerical modelling integrated with field observations and analytical data of the 2021 Cumbre Vieja eruption improves understanding of eruption dynamics at mafic volcanoes. *J Geophys Res Solid Earth* **130**, e2024JB029692 (2025).
6. Llewellin, E. W., Mader, H. M. & Wilson S. D. R. The constitutive equation and flow dynamics of bubbly magmas. *Geophysical research letters* **29**, 23-1 (2002).
7. Mader, H. M., Llewellin E. W., & Mueller S. P. The rheology of two-phase magmas: A review and analysis. *J. Volcanol. Geotherm. Res*. **257,** 135-158 (2013).
8. La Spina, G. et al. Conduit dynamics of highly explosive basaltic eruptions: The 1085 CE Sunset Crater sub-Plinian events. *J. Volcanol. Geotherm. Res*. **387**, 106658 (2019).
9. Bonechi, B. et al. Magma residence time, ascent rate and eruptive style of the November ash-laden activity during the 2021 Tajogaite eruption (La Palma, Spain). *Volcanica* **7**, 51–65 (2024).
10. Bonadonna, C. et al. Physical Characterization of Long-Lasting Hybrid Eruptions: The 2021 Tajogaite Eruption of Cumbre Vieja (La Palma, Canary Islands). *J. Geophys. Res. Solid Earth* **127**, e2022JB025302 (2022).
