# Supplementary material for: Superheating in mafic magmas controls clinopyroxene nucleation delay and magma ascent dynamics
Source: Nat Commun. 2026 Jun 8;17:4962. doi: 10.1038/s41467-026-73352-1 (PMC13247257; doi:10.1038/s41467-026-73352-1)
Supplement: Supplementary file 2 — Description of Additional Supplementary Files [file 41467_2026_73352_MOESM2_ESM.pdf]

Description of Additional Supplementary Files

Supplementary Data 1. Experimental conditions and phase assemblage of *in situ* and *ex situ* view experiments

Supplementary Data 2. Number of crystals per area for *in situ* and *ex situ* view experiments

Supplementary Data 3. Clinopyroxene growth rates for *in situ* and *ex situ* view experiments

Supplementary Data 4. Chemical composition of natural starting material
