# Supplementary material for: Superheating in mafic magmas controls clinopyroxene nucleation delay and magma ascent dynamics
Source: Nat Commun. 2026 Jun 8;17:4962. doi: 10.1038/s41467-026-73352-1 (PMC13247257; doi:10.1038/s41467-026-73352-1)
Supplement: Supplementary file 4 — Transparent Peer Review file [file 41467_2026_73352_MOESM4_ESM.pdf]

nature portfolio

Peer Review File

Superheating in mafic magmas controls clinopyroxene nucleation delay and magma ascent dynamics

Corresponding Author: Dr Barbara Bonechi

This file contains all reviewer reports in order by version, followed by all author rebuttals in order by version.

Version 0:

Reviewer comments:

Reviewer #2

(Remarks to the Author)
Review of "The role of superheating on clinopyroxene nucleation delay in mafic magmas: implications for pre-eruptive crystal content and magma ascent dynamics"

Summary
This study investigates the impact of superheating on clinopyroxene nucleation in mafic magmas from the 2021 Tajogaite eruption (La Palma, Spain). Using synchrotron X-ray microtomography to observe crystallization in real time, the authors demonstrate that superheating dissolves pre-existing nuclei and delays clinopyroxene nucleation. Numerical modelling suggests that the crystallization timescale has a strong influence on both ascent rate and eruption rate, primarily through its effect on magma viscosity. Overall, the study emphasizes the significance of pre-eruptive thermal history in magma ascent dynamics.

General remarks
The manuscript is generally well-written and clearly presented. The novel in-situ experiments enable real-time observation of magma crystallization, not at atmospheric pressure, but under crustal pressure and water-saturated conditions. This technique represents a valuable foundation for investigating crystallization kinetics under realistic conditions relevant to volcanic eruptions. The study also nicely documents sample heterogeneity using Raman microscopy and differential scanning calorimetry. Because magma ascent dynamics are highly sensitive to crystal content, these in-situ and ex-situ experiments provide essential insights into how pre-eruptive superheating influences eruption behaviour.

I recognize the significance of the new experimental technique. However, I am not entirely convinced by the authors' claim that nucleation delay due to superheating was primarily responsible for the shift in eruptive style during the 2021 Tajogaite eruption. The manuscript has several issues:
•The role of undercooling is not adequately considered, leading to an overestimation of superheating effects.
•The characteristic crystallization timescale used in the numerical modelling does not reflect the experimental results.
•Crystallization kinetics of phases other than clinopyroxene are insufficiently quantified.

I encourage the authors to address these points to support their main claim. The study's value lies especially in the development of the experimental technique, but stronger evidence is needed to elucidate the effect of superheating on eruption dynamics. I recommend a major revision.

Major issues
1. Undercooling vs. superheating
My main concern is the different degrees of undercooling between the ex-situ and in-situ experiments. Nucleation and growth rates are generally dependent on the degree of undercooling. In the ex-situ experiment, undercooling was constant (ΔT_{uc} = 12 °C), whereas in the in-situ experiment it increased with time. Thus, crystallization kinetics were controlled by both undercooling and superheating. However, the authors ascribe the higher nucleation and growth rates in the in-situ experiments only to superheating (lines 242–243). It seems more likely that the higher degree of undercooling in the in-situ experiments explains these rates.

I recommend plotting number density and growth rate as functions of undercooling (modifying Fig. 6 accordingly). How

sensitive are these parameters to undercooling? Do the experimental variations reflect superheating alone, or also undercooling?

2. Integration of experimental results into numerical modelling
The authors claim that the experimental results were integrated into the numerical model (lines 110–111), but the link is unclear. Specific issues include:
•The definition of the characteristic crystallization time (τ_c) is not provided. A mathematical formulation relating τ_c to crystallinity should be included.
•The high growth and nucleation rates in the in-situ experiments likely result from large undercooling rather than superheating (see Major issue 1). The authors should provide a clear explanation of why superheating affects the rate of crystallinity increase.
•The model uses two arbitrary τ_c values (10^3 and 10^5 s). For simulations relevant to the Tajogaite eruption, τ_c should be derived from the experimental data.
•The current numerical results illustrate only general trends of τ_c, which are well demonstrated in previous studies. This study provides limited insight into the Tajogaite eruption.
These issues must be addressed to convincingly connect the experiments with the numerical model.

3. Other crystal phases
The study focuses exclusively on clinopyroxene, but other crystal types were observed. The rationale for this focus is unclear.
•Please provide phase abundances for all experimental products. Was clinopyroxene the dominant phase?
•How did nucleation delays of other phases vary with superheating?
•Plagioclase appeared only in in-situ experiments, while chromite appeared only in ex-situ experiments. Why? Could sample preparation or pressure conditions have influenced crystallization kinetics? If so, the different nucleation delay between in-situ and ex-situ experiments cannot be ascribed solely to superheating.

Minor issues
Figures 1–3 and 6: Ensure consistent definition of time. Some figures use the onset of heating as time zero, while others use the onset of tomography. For example, in Fig. 6b, why does the growth rate start at ~410 min if nucleation began after 8 hours (line 155, Fig. 6a)?
Figure 2: Clarify what “time*” represents.
Figure 6: Explain how error bars were determined. Why are there no error bars for the red circles in panel A?
Figure 7: Does the mixture viscosity account for bubbles?
Line 106: Add justification for focusing on clinopyroxene.
Line 153: Define ΔT_{uc}^{final}.
Lines 221–224: Specify which supplementary data are being referred to. The claim that crystals in the in-situ view are smaller than in the ex-situ view was not evident from Figs. A1–A5 .
Lines 227–228: Clarify how crystal number density was measured. Did you use tomography or BSE images? What was the minimum detectable crystal size? This information is missing from the methods section.
Lines 242–243: As noted above, both nucleation and growth rates were controlled by undercooling. The supposed inverse relationship with superheating is unclear.
Lines 739–741: Indicate which experimental run was used for the in-situ data.

Reviewer #3

(Remarks to the Author)
Dear Editor, Dear Authors,
I have now reviewed the manuscript from Bonechi et al. “The role of superheating on clinopyroxene nucleation delay in mafic magmas: implications for pre-eruptive crystal content and magma ascent dynamics”.

The manuscript describes the results of crystal nucleation experiments performed both in-situ and ex-situ, finding that increasing overheating increases the delay for crystal nucleation, which they show impacts on eruptive dynamics.

I think this is a very good study, however there are various issues that have to be addressed.
The most important for me is the framing of the study. When I read the manuscript the first time, I thought almost until the very end that the authors were considering models in which magma is reheated by magma injection above its liquidus temperature, which I think has basically no applications to nature (I will explain later why). I think the problem starts at Lines 60-61 (….”for example through the injection of mafic magma). First, I explain why reheating significantly above liquidus temperature is essentially not possible in nature (especially when the main application of this study is to the 2021 La Palma eruption):

Reheating magma above its liquidus temperature in nature is extremely difficult and thus the “overheating by magma injection” scenario might only apply to the injection of large masses of basalt into a chemically evolved reservoir, which would significantly limit the applications of your study and make it less impactful (which would be a shame). My suggestion would be to frame your experiments to consider decompression-induced overheating

Consider for instance La Palma: magma produced in the mantle is extracted (by definition) at the solidus temperature of the

mantle (and the liquidus temperature of the basalt). If this magma is injected in a reservoir of the same magma that has just cooled and crystallised (i.e. the liquidus temperature has not changed if water was not removed from the system), the injection of the same basalt extracted from the mantle cannot results into an increase of temperature above liquidus (as the liquidii of the two magmas are the same). And even to bring back the temperature of the magma in the reservoir to values close to its liquidus temperature, would require the injection of a mass of magma that is much much larger than the mass of magma within the reservoir.
If the resident magma has lost water, but its chemical composition did not evolve significantly, it is not possible to increase the temperature above its liquidus, injecting its parental magma.

Now, I explain why this is not only a semantic problem and reframing the study on decompression induced-overheating requires some attention to avoid internal contradiction of the arguments presented by the authors.
The hypothesis proposed by the authors to explain the impact of overheating on nucleation is summarised at Lines 204-208: “Combined observations from DSC and in situ view
experiments conducted near the liquidus suggest that incipient crystalline clusters or nanolites, possibly formed during the initial heating ramp, may act as heterogeneous nucleation sites and significantly reduce incubation times. Conversely, superheating above the liquidus leads to the dissolution of such structures, yielding a more structurally homogeneous and disordered melt. This homogenization increases the thermodynamic and kinetic barriers to nucleation, thereby extending the nucleation delay by requiring greater undercooling to re-establish favourable conditions for crystal formation”. If the results of the experiments are framed in this way, the hypothesis stands only if magma is reheated above its liquidus temperature, but it is not granted that this model would apply to overheating occurring during the adiabatic (or quasi-adiabatic) ascent of magma, which does not imply a supply of energy to the system, but actually a decrease of heat associated to the work done for the expansion of the melt to take place.

Something I disagree with. The authors state at Lines 150-152 that: “The different pressures of the in situ and ex situ view experiments do not significantly affect the comparability of the experimental results, since pressure has a negligible effect on crystallization kinetics”.
Actually, your glasses are fully degassed, thus an increase of pressure will result in an increase of liquidus temperature of the material. This means that the liquidus temperature should be higher for the experiments performed in ex-situ conditions with respect to those performed in-situ. Under these conditions, if the initial temperature is the same, the degree of undercooling is lower for the ex-situ experiments than for the in-situ ones. Hammer (2008) describes the effect of undercooling on crystal nucleation delay: (cit. page 35) " Striking contrasts between quasi-continuous decompression and single-step decompression run products underscore the importance of decompression history in controlling crystal texture (Fig. 15). For example, samples decompressed to final pressures >40 MPa in multiple steps were much less likely to reach equilibrium crystallinity than samples decompressed in a single step. Because crystallization during each decompression step brings the system closer to equilibrium, stepwise decompression produces smaller instantaneous ΔT_{eff} than is typical in the single-step decompressions. That the slowly-decompressed samples were less likely to achieve chemical equilibrium than their rapidly-decompressed counterparts is interpreted to reflect this generally lower degree of undercooling in the multistep run throughout the experiment. This observation runs contrary to conventional wisdom, which would suggest slow decompression is more likely to promote chemical equilibration than rapid decompression."
The multiple steps decompression experiments in Hammer 2008 are equivalent to smaller degrees of undercooling in your experiments (i.e. the ex-situ experiments), and therefore according to Hammer 2008, it is expected that nucleation is delayed in your higher-pressure experiments. Perhaps this is something the authors can use to reframe their study?

I added some further minor comment sin the annotated version of the manuscript.
Again, I think this is a very important study, and I hope the authors will find my comments useful as I would like to see this study published.

Best Regards,

Luca Caricchi

REFERENCE
Hammer, Julia E. “Experimental Studies of the Kinetics and Energetics of Magma Crystallization.” Reviews in Mineralogy and Geochemistry 69, no. 1 (2008): 9–59. https://doi.org/10.2138/rmg.2008.69.2.

Reviewer #5

(Remarks to the Author)
The manuscript “The role of superheating on clinopyroxene nucleation delay in mafic magmas: implications for pre-eruptive crystal content and magma ascent dynamics” by Bonechi et al. presents an experimental and numerical modeling study on the reheating of magma above the clinopyroxene liquidus, followed by subsequent cooling. The authors argue that such reheating alters crystallization behavior because the dissolution of pre-existing crystals promotes homogeneous crystallization, which operates on significantly longer timescales compared with heterogeneous crystallization. This mechanism has implications for magma ascent and eruption style, as reduced crystallization results in lower melt viscosity and potentially higher ascent velocities.

Overall, this is an interesting and well-written study with a clear structure and a logical flow. The research question is highly relevant, as the pre-eruptive processes governing the effusive–explosive transition, and eruption style more broadly, remain poorly constrained. Any contribution that refines our understanding of such processes is therefore valuable.

I find the experimental component, involving superheating and subsequent crystallization, to be well designed and presented. I have no major comments on that section.
The main concerns I have, relate to magma ascent modeling. The simulations only model the effect of varying crystallization timescales rather than explicitly modeling superheating. Previous studies (e.g., Kozono & Koyaguchi, 2012) have already demonstrated how crystallization kinetics influence eruption style, so the modeling results as presented, do not appear to introduce fundamentally new insights.

Specific comments
• The 2021 Cumbre Vieja eruption has been extensively studied in recent years. I expected a more detailed synthesis of available data (e.g., Ubide et al., 2023; Romero et al., 2022; Dayton et al., 2024, etc) regarding eruption styles (fountaining, effusion, etc.) and their correlation with pre-eruptive temperatures, crystal contents, and related parameters, rather than the broad discussion presented in lines 302–312. Since the authors propose that superheating may have influenced eruption style, the manuscript would benefit from more concrete observational constraints supporting this hypothesis.
• The study focuses on clinopyroxene crystallization. While clinopyroxene was indeed the dominant crystalline phase during the 2021 eruption, plagioclase, Fe–Ti oxides, and amphibole were also present. How are these phases treated—or neglected—in the simulations, and what are the implications of excluding them?
• If one simulation case is intended to represent superheating, why are the initial temperatures identical in both cases? Similarly, why is the initial crystal content the same? Superheating should imply partial or complete dissolution of crystals prior to ascent, so why the same initial crystal content (and temperature) for both?. Although the authors state that the key factor is the kinetic slowdown caused by melt homogenization, the conduit simulations reproduce results already shown in previous works (e.g., Arzilli et al., 2019), without adding something new regarding the different consequences of superheating.
Other comments
• In the title and subtitles, the phrase "… implications for pre-eruptive crystal content" could be misleading, as it may be interpreted as referring to initial crystal content in the reservoir. A more accurate phrasing might be "implications for ascent crystallization."
• Figures 3 and 4 could be moved to the Supplementary Material, as they are not essential for the main narrative.

Conclusion
In summary, this is a potentially strong manuscript that merits publication. However, the section on implications for ascent dynamics requires further refinement. In particular, demonstrating how superheating influences ascent dynamics not only in crystallization kinetics but also in initial crystal content and temperature, and incorporating more detailed observational data from the 2021 Cumbre Vieja eruption that supports the main hypothesis, would substantially strengthen the authors' conclusions.

Version 1:

Reviewer comments:

Reviewer #2

(Remarks to the Author)
This is my second review of the manuscript. I am satisfied with the way my comments and those of the other reviewers have been addressed. I have no further comments and recommend acceptance of the manuscript.

Reviewer #3

(Remarks to the Author)
Dear Authors,
I appreciate the effort to perform the calculations linked to the new Figure 6 and I think this could potentially be a nice addition to the paper.
However, in the current format the results are not correctly presented and discussed.
At lines 411-415 the authors state: "Our results indicate that superliquidus conditions are favoured under more reduced conditions and at higher H2O contents, as liquidus temperatures decrease under these conditions. The modelled scenarios here define distinct superheating regimes governed by different degrees of disequilibrium crystallisation during ascent, with liquidus positions modulated by water content and redox state". The issue here is that the initial temperature of the P-T path is always the same. This is incorrect as the magma liquidus' temperature is linked to its water content and initial oxygen fugacity (initial because the oxygen fugacity of magmas will change upon ascent, see for example Burgisser and Scaillet , 2007 Nature). For instance, if a magma contains more water its initial liquidus temperature will be lower so the initial point of the curves at lower temperatures should be lower. Both curves in Figure 6 will always lead to crystallisation because their slope is lower than the slope of the liquidii for magmas with different water contents.

Water content and (potentially) oxygen fugacity will impact the evolution of the magma's crystal content only if they affect the slope of the liquidus curves with respect to the slope of the P-T path considered.

I urge the authors to take care of this section and Figure 6.

Best Regards,

Luca

Reviewer #5

(Remarks to the Author)
I thank the authors for the substantial revision of the manuscript, “The role of superheating on clinopyroxene nucleation delay in mafic magmas: Implications for pre-eruptive crystal content and magma ascent dynamics.” Overall, I find the revised version significantly improved relative to the original submission. In the first round, my main concerns were focused primarily on the magma ascent modelling rather than on the experimental component, which I already considered strong. In this revised version, the modelling section is clearer, better motivated, and more closely connected to the experimental results than before.

Most importantly, the authors have now modified the conduit model to account explicitly for nucleation delay, rather than relying only on contrasting crystallisation timescales. This is a meaningful improvement and addresses one of my main concerns from the previous round. The revised manuscript now uses nucleation delays derived from the experiments (20 min versus 8 h) as end-member inputs in the modelling framework, which provides a much more direct conceptual connection between the experiments and the numerical exploration.

I also appreciate that the revised manuscript now makes much clearer that the purpose of the modelling is not to reconstruct the 2021 Tajogaite eruption in detail, but rather to use Tajogaite as a representative mafic case study to explore a more general physical mechanism. This clarification improves the framing substantially. In the original version, the application to Tajogaite felt too specific relative to the level of constraint provided by the model. The revised text is more appropriately framed in this respect.

The discussion of other crystalline phases is also improved. The authors now explain more explicitly that the simulations track total crystal fraction rather than phase-specific crystallisation, and that clinopyroxene is treated as a representative proxy for the onset of crystallisation because it is the dominant early phase in this system and the one constrained experimentally. I find this acceptable within the scope of the study.

That said, I still have one remaining concern, which is mostly about emphasis and interpretation, rather than about the core validity of the study. Even in the revised version, the modelling remains an illustrative, end-member-based exploration, not a quantitatively constrained reconstruction of Tajogaite. I therefore encourage the authors to keep the interpretation carefully phrased throughout, especially where the manuscript suggests that superheating may explain specific eruptive transitions or variations in eruptive style during the 2021 eruption. In my view, the study now demonstrates a plausible and potentially important mechanism, but not yet a uniquely constrained explanation for Tajogaite itself.

Related to this point, I think the manuscript would benefit from one final pass to ensure that the strongest claims are consistently aligned with what the model actually shows. The real strength of the study is that it demonstrates how experimentally motivated nucleation delays can have major consequences for ascent dynamics under certain initial conditions and timescales. This is already a valuable result. It does not need to be overstated as a specific explanation for the natural evolution of Tajogaite. The authors themselves now acknowledge that pre-eruptive thermodynamic conditions primarily control magma ascent dynamics, and that nucleation delay becomes particularly important when ascent timescales are comparable to nucleation-delay timescales. I agree with that formulation and suggest that it should guide the tone of the final discussion.

Recommendation

Overall, I consider that the authors have addressed my main concerns, and that the revised manuscript is now suitable for publication after minor revision.

Remaining minor points
I suggest that the authors strengthen the discussion of the Tajogaite case study by briefly reporting the observed range of eruptive or mass discharge rates during the 2021 eruption, and clarifying whether the modelled contrast between the two end-member scenarios is comparable in magnitude to the natural variations documented during Tajogaite. This would provide a more concrete basis for evaluating whether the proposed role of superheating-induced nucleation delay is consistent with the scale of variability observed in nature. At present, the manuscript suggests that superheating may have contributed to the variability in eruptive style and intensity, but this link would be more convincing if the authors explicitly compared the modelled changes with the natural range of eruptive-rate variability inferred for Tajogaite.

Open Access This Peer Review File is licensed under a Creative Commons Attribution 4.0 International License, which permits use, sharing, adaptation, distribution and reproduction in any medium or format, as long as you give appropriate credit to the original author(s) and the source, provide a link to the Creative Commons license, and indicate if changes were made.
In cases where reviewers are anonymous, credit should be given to 'Anonymous Referee' and the source.
The images or other third party material in this Peer Review File are included in the article's Creative Commons license, unless indicated otherwise in a credit line to the material. If material is not included in the article's Creative Commons license and your intended use is not permitted by statutory regulation or exceeds the permitted use, you will need to obtain permission directly from the copyright holder.
To view a copy of this license, visit https://creativecommons.org/licenses/by/4.0/

Response to reviewers' comments for NCOMMS-25-57465

Responses to reviewers' comments and changes in the main text are reported in light blue

Reviewer #2

General remarks

The manuscript is generally well-written and clearly presented. The novel in-situ experiments enable real-time observation of magma crystallization, not at atmospheric pressure, but under crustal pressure and water-saturated conditions. This technique represents a valuable foundation for investigating crystallization kinetics under realistic conditions relevant to volcanic eruptions. The study also nicely documents sample heterogeneity using Raman microscopy and differential scanning calorimetry. Because magma ascent dynamics are highly sensitive to crystal content, these in-situ and ex-situ experiments provide essential insights into how pre-eruptive superheating influences eruption behaviour.

I recognize the significance of the new experimental technique. However, I am not entirely convinced by the authors' claim that nucleation delay due to superheating was primarily responsible for the shift in eruptive style during the 2021 Tajogaite eruption.

The manuscript has several issues:

- *The role of undercooling is not adequately considered, leading to an overestimation of superheating effects.*
- *The characteristic crystallization timescale used in the numerical modelling does not reflect the experimental results.*
- *Crystallization kinetics of phases other than clinopyroxene are insufficiently quantified.*

I encourage the authors to address these points to support their main claim. The study's value lies especially in the development of the experimental technique, but stronger evidence is needed to elucidate the effect of superheating on eruption dynamics. I recommend a major revision.

We thank the reviewer for the thorough comments. Answers to all issues/comments can be found in the following replies.

Major issues

1. Undercooling vs. superheating

My main concern is the different degrees of undercooling between the ex-situ and in-situ experiments. Nucleation and growth rates are generally dependent on the degree of undercooling. In the ex-situ experiment, undercooling was constant (ΔT_{uc} = 12 °C), whereas in the in-situ experiment it increased with time. Thus, crystallization kinetics were controlled by both undercooling and superheating. However, the authors ascribe the higher nucleation and growth rates in the in-situ experiments only to superheating (lines 242–243). It seems more likely that the higher degree of undercooling in the in-situ experiments explains these rates.

I recommend plotting number density and growth rate as functions of undercooling (modifying Fig. 6 accordingly). How sensitive are these parameters to undercooling? Do the experimental variations reflect superheating alone, or also undercooling?

We thank the reviewer for this insightful comment. We agree that undercooling plays a fundamental role in crystallisation kinetics. We carefully examined our experimental data to disentangle the effects of undercooling and superheating.

In the *in situ* experiments, undercooling increases during the experiment as a consequence of the continuous cooling ramp, which reaches lower temperatures (1020–1080 °C) than the *ex situ*

experiments (1100 °C). The *ex situ* experiments indeed experience a nearly constant degree of undercooling (ΔT_{uc} ~12 °C). To account for this difference, we recalculated the degree of undercooling corresponding to each tomographic slice used to extract crystal number density and growth rates. These values are now reported in Supplementary Tables S2–S3.

Following the reviewer's suggestion, we have also replotted both number of crystals and growth rates as a function of undercooling and modified Figure 6 accordingly (now Fig. 4 in the revised version). The revised plots show that crystal number density (the number of crystals per area) increases with increasing undercooling (see figure below, plot B), confirming that undercooling exerts a first-order control on nucleation.

Undercooling remains the primary thermodynamic driving force for crystallisation, controlling nucleation and growth and strongly influencing crystal number density, nucleation rate, and growth rate once crystallisation begins. Greater undercooling generally promotes higher nucleation rates and faster crystal growth.

However, at comparable degrees of undercooling, crystallisation kinetics can still be significantly affected by prior superheating (Billon et al., 2026). Extensive superheating can modify the melt structure, for example, by dissolving pre-existing clusters or structural heterogeneities, thereby delaying nucleation upon cooling into subliquidus conditions. As a result, even under similar undercooling, larger superheating may reduce the effective nucleation rate and crystal number density, and alter subsequent growth behaviour.

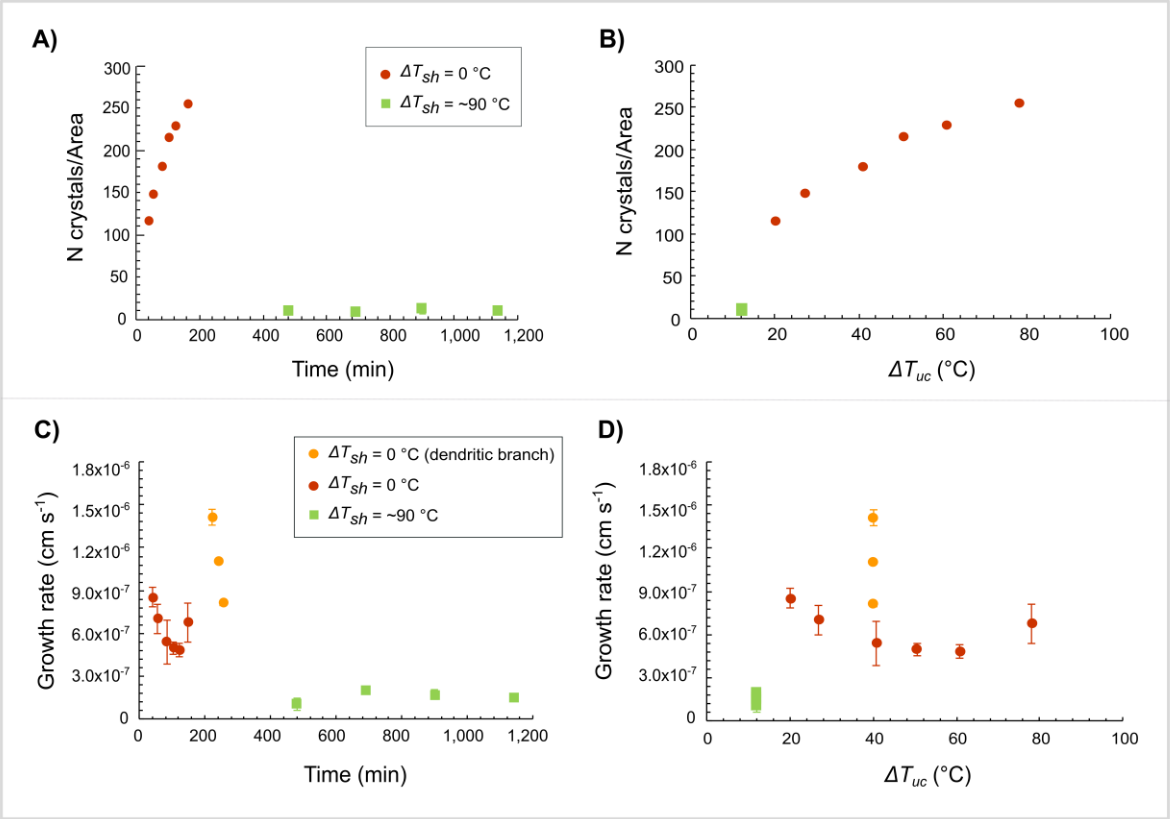

In summary, while undercooling provides the fundamental thermodynamic driving force for crystallisation at subliquidus conditions, the melt's superheating history exerts a critical kinetic influence. Consequently, the observed crystallisation behaviour reflects the combined effects of undercooling and superheating, explaining the higher number of crystals observed in the *in situ* experiments compared with the *ex situ* ones.

This effect is now explicitly acknowledged and discussed in the revised manuscript (Lines 264-288). In addition, previously misreported area values for the *in situ* measurements have been corrected (Table S2), and figures updated accordingly.
Regarding crystal growth, growth rates generally decrease with increasing undercooling in the *in situ* LP1-CC05 experiment (see red dots in the figure above), consistent with enhanced nucleation competing for available melt, as observed in previous studies (Masotta et al., 2020). We reported this behaviour in the revised manuscript (Lines 294-298). Importantly, the superheated (*ex situ*) experiments (green squares) systematically exhibit lower number of crystals per area and growth rates than the *in situ* experiments even at comparable degrees of undercooling. This behaviour highlights the key role of superheating in promoting the dissolution of pre-existing nuclei and melt homogenisation, thereby suppressing both nucleation efficiency and subsequent crystal growth (revised manuscript, Lines 300-307).

2. Integration of experimental results into numerical modelling
The authors claim that the experimental results were integrated into the numerical model (lines 110–111), but the link is unclear.

The reviewer is right, the word "integrating" was not appropriate in this context. Indeed, in the previous version of the manuscript, the numerical simulations were used to infer the role of a prolonged nucleation delay on magma ascent dynamics, even though nucleation delay was not explicitly modelled. To overcome this limitation, we now modified the numerical code to explicitly include the nucleation delay (see supplementary Data). While computing the numerical solution, the code now calculates magma ascent time at each depth integrating magma ascent velocity. Crystallisation is then neglected until ascent time is equal to the nucleation delay, and only from that depth until the vent of the conduit crystallisation is permitted (following the transport equation for the evolution of the crystal volume fraction). Following this development, we now directly compare the numerical simulations obtained with the nucleation delays resulting from the experiments at different degree of superheating (i.e., 20 minutes for no superheating, and 8 hours for ~90°C of superheating). Accordingly, we have revised the manuscript (Lines 335-353), updated the Supplementary Data, and modified Figures 5 and S4.

Specific issues include:
•The definition of the characteristic crystallization time (τ_c) is not provided. A mathematical formulation relating τ_c to crystallinity should be included.

The characteristic time of crystallisation controls how quickly the crystallisation process occurs due to a perturbation of the thermodynamic conditions. Arzilli et al. (2019) and La Spina et al. (2016) indicate that a characteristic time of 1000 s is reasonable for mild explosive activity in low viscosity basaltic magmas, whereas a shorter characteristic time (up to tens of seconds) may be achieved for highly explosive eruptions (Plinian or sub-Plinian) due to very large undercooling (> 100 °C). The 2021 Tajogaite eruption was a long-lasting low viscosity mafic eruption, involving effusive and explosive activity from multiple vents with modest mass eruption rates (from 10^3 – 10^5 kg/s). The high magmatic temperature for the Tajogaite eruption are not likely to generate very large undercooling, so a fast crystallisation (and thus a characteristic time of 10 s) is not realistic. Furthermore, Biagioli et al. (2025) also performed numerical simulations of the 2021 Tajogaite eruption and they considered a characteristic time of crystallisation of 1000 s. For these reasons, we considered in our simulations a constant characteristic time of 1000 s. This has been now clarified in the supplementary information (Supplementary Data).

In the previous version of the manuscript, we also considered a characteristic time of 10^5 s, which was done to mimic the effect of a very long nucleation delay (effectively neglecting crystallisation during ascent). Now that we have properly included the nucleation delay in our model, we removed the simulation performed with a characteristic time of 10^5 s.

Moreover, we remark here that it is not the purpose of this work to define a mathematical formulation of the characteristic time of crystallisation. To derive a proper description of τc as function of the thermodynamic conditions more experiments would be needed. The purpose of this work is, instead, to investigate the effect of superheating and of nucleation delay on magma ascent dynamics in mafic magmas.

•*The high growth and nucleation rates in the in-situ experiments likely result from large undercooling rather than superheating (see Major issue 1). The authors should provide a clear explanation of why superheating affects the rate of crystallinity increase.*

We thank the reviewer for this comment. The role of undercooling is addressed in detail in our response to Comment #1 ("*Undercooling vs. superheating*").

Our experiments further demonstrate that superheating independently affects crystallisation kinetics by controlling the availability of pre-existing nuclei prior to crystallisation. Prolonged residence at superliquidus conditions promotes efficient dissolution of inherited nuclei and enhances melt homogenisation, thereby reducing the number of active nucleation sites once subliquidus conditions are reached. As a consequence, even at comparable degrees of undercooling (ΔT at the onset of nucleation; Table S1), the superheated (*ex situ*) experiments systematically display lower nucleation densities and slower crystal growth rates than the *in situ* experiments (revised Figure 4).

We have clarified this mechanism in the revised manuscript (Lines 264–288), emphasising that undercooling primarily governs the rate of crystal nucleation and growth once crystallisation begins, whereas superheating determines the initial structural state of the melt and thus the efficiency of crystallinity increase during subsequent cooling.

•*The model uses two arbitrary τ_c values (10^3 and 10^5 s). For simulations relevant to the Tajogaite eruption, τ_c should be derived from the experimental data.*

As we indicated above, we previously considered a characteristic time of 10^5 s to mimic the effect of a very long nucleation delay. Now that we have properly included the nucleation delay in our model, we removed the simulation performed with a characteristic time of 10^5 s.

Furthermore, the 2021 Tajogaite eruption was a low viscosity mafic eruption with modest mass eruption rates (from 10^3 – 10^5 kg/s). For this reason, as indicated by La Spina et al. (2016) and Arzilli et al. (2019) we considered in our simulations a constant characteristic time of 1000 s.

To derive a proper description of τc as function of the thermodynamic conditions we would need more experiments, but this is not the purpose of this work. We aim to investigate the effect of superheating and of nucleation delay on magma ascent dynamics in mafic magmas. For this reason, we now implemented nucleation delay in the conduit code, and we performed numerical simulations using corresponding values for nucleation delays constrained by the experiments.

•*The current numerical results illustrate only general trends of τ_c, which are well demonstrated in previous studies. This study provides limited insight into the Tajogaite eruption. These issues must be addressed to convincingly connect the experiments with the numerical model.*

As we indicated above, we removed the simulation performed with a characteristic time of 10^5 s, and performed numerical simulations considering different nucleation delays, as constrained by experimental results.

We remark that our aim is not to model the 2021 Tajogaite eruption. This eruption has been considered as a test case scenario due to the very high temperatures involved, which may have been affected by superheating and a very large nucleation delay.

3. Other crystal phases

The study focuses exclusively on clinopyroxene, but other crystal types were observed. The rationale for this focus is unclear.

•Please provide phase abundances for all experimental products. Was clinopyroxene the dominant phase?

Clinopyroxene is the dominant phase in our experiments, which is why this study focuses on its crystallisation kinetics. Moreover, in the *in situ* experiments, clinopyroxene can be reliably identified owing to its sufficient density contrast with the melt and its larger crystal size relative to the other phases. We have clarified the rationale for focusing on clinopyroxene in the revised manuscript (Lines 135-138). Other crystal phases, such as olivine, plagioclase, and oxides, are also present as shown qualitatively in the Supplementary Data (post-mortem images of *in situ* and *ex situ* samples). However, because they cannot be clearly resolved in real time due to their low density contrast and/or crystal sizes below our detection limit, their abundances cannot be quantified in the *in situ* experiments. This does not affect the main focus of the study on clinopyroxene nucleation delay.

•How did nucleation delays of other phases vary with superheating?

In our *in situ* experiments, other phases are not clearly detectable during the experiment because they have a lower density contrast with the melt and/or crystal sizes below our detection limit. As a result, we cannot determine their nucleation times or delays. Therefore, nucleation delays for phases other than clinopyroxene cannot be quantified from our current data.

•Plagioclase appeared only in in-situ experiments, while chromite appeared only in ex-situ experiments. Why? Could sample preparation or pressure conditions have influenced crystallization kinetics? If so, the different nucleation delay between in-situ and ex-situ experiments cannot be ascribed solely to superheating.

Plagioclase crystallisation in our system occurs at lower temperatures (below ~1080 °C), which are reached during the continuous cooling applied in the *in situ* experiments, but not in the *ex situ* experiments, which were held at ~1100 °C. In addition, the *in situ* experiments were performed at low pressure (≤ 20 MPa), whereas the *ex situ* experiments were conducted at higher pressure (275 MPa), conditions that are known to suppress plagioclase stability in this tephritic melt (Fabbrizio et al., 2023).

Conversely, chromite and other oxide phases are expected to crystallise early at high temperature and pressure and are therefore readily observed in the *ex situ* experiments. While oxides are likely also present in the *in situ* experiments, their very small size places them below the spatial resolution of the imaging system used, preventing their unambiguous detection.

Therefore, the contrasting appearance of these minor phases reflects differences in experimental temperature–pressure paths and detection limits, rather than differences in crystallisation kinetics induced by sample preparation or superheating. Importantly, our analysis of nucleation delay and

crystallisation kinetics is focused on clinopyroxene, which is the dominant phase in all experiments (*in situ* and *ex situ*) and crystallises over a comparable temperature range. This allows for a robust and internally consistent comparison between *in situ* and *ex situ* experiments, isolating the effect of superheating on clinopyroxene nucleation and growth.

Minor issues

Figures 1–3 and 6: Ensure consistent definition of time. Some figures use the onset of heating as time zero, while others use the onset of tomography. For example, in Fig. 6b, why does the growth rate start at ~410 min if nucleation began after 8 hours (line 155, Fig. 6a)?

We thank the reviewer for the comment. There was an error in the reported figure 6b. The original plot starts at 480 min. We corrected the image (Fig. 4C).

Figure 2: Clarify what "time" represents.*

We thank the reviewer for pointing this out. In Figure 2, "time*" represents the time elapsed from the start of the tomographic recording, which begins at approximately 960 °C. We have clarified this in the figure caption in the revised manuscript (Lines 872-874).

Figure 6: Explain how error bars were determined. Why are there no error bars for the red circles in panel A?

We thank the reviewer for this comment. In Figure 6 (now Fig. 4), error bars represent the standard deviation of measurements obtained from multiple crystals used for growth rate calculations within each experiment; panel C-D). We added this information in the revised text (Lines 882-883).

The nucleation density values (panel A-B, both *in situ* and *ex situ*) are actual counts of crystals in the analysed area, and no standard deviation was calculated, and error bars are not appropriate. The small error bar visible with the *ex situ* points in the originally submitted figure was a residual artefact from a previous version of the plot and does not reflect the measurement. We have corrected the figure in the revised manuscript.

Figure 7: Does the mixture viscosity account for bubbles?

In the simulations presented in the original version of the manuscript the viscosity of magma was calculated as the viscosity of the melt multiplied by a factor related to the presence of crystals. For completeness, we now included also the relative viscosity due to the presence of bubbles, as described in Llewellin et al. (2002), Mader et al. (2013) and La Spina et al. (2019). This has been now clarified in the supplementary information (Supplementary Data).

Line 106: Add justification for focusing on clinopyroxene.

We added a justification for focusing on clinopyroxene in the revised manuscript (Lines 135-138). Also see explanation given at point 3 above.

Line 153: Define ΔT_{uc}^{final}.

ΔT_{uc}^{final} corresponds to ΔT_{uc} as defined and explained in line 142. To avoid confusion, we have corrected the notation throughout the manuscript.

Lines 221–224: Specify which supplementary data are being referred to. The claim that crystals in the in-situ view are smaller than in the ex-situ view was not evident from Figs. A1–A5.

We thank the reviewer for this comment. The comparison of crystal sizes between the *in situ* and *ex situ* view experiments refers to the Supplementary Data file (PDF), which includes observations from recovered samples and additional figures, including BSE images of the experimental products. To make this difference clearer, we have revised the Supplementary Data by adding a new Figure A5, which shows in greater detail the phases crystallised in the *ex situ* view experiments and highlights the larger clinopyroxene crystals. In addition, we have added an additional scale bar to Figure A6 for the *in situ* view experiments to allow a clearer visualisation of crystal dimensions. We also revised the text in both the Supplementary Data and the main manuscript (Lines 251-253) to explicitly report the measured crystal lengths (up to ~200 μm in the *in situ* view experiments and up to ~400 μm in the *ex situ* view experiments), making the size difference between the two experimental approaches more explicit and easier to evaluate.

Lines 227–228: Clarify how crystal number density was measured. Did you use tomography or BSE images? What was the minimum detectable crystal size? This information is missing from the methods section.

We thank the reviewer for this comment. The number of crystals per area was measured using different imaging techniques depending on the experimental approach. For the *in situ* experiments, crystal counts were performed on reconstructed synchrotron X-ray microtomographic slices, whereas for the *ex situ* experiments, the number of crystals was measured on BSE images of polished sections. Tomographic slices were used for *in situ* experiments because they provide time-resolved information during crystallisation, while *ex situ* measurements necessarily rely on BSE images of the final, post-experiment samples.

For the tomographic datasets, the minimum detectable crystal size is determined by the spatial resolution and the phase-contrast imaging mode, which enables the observation of crystals with dimensions close to the voxel size. Based on these constraints, the smallest crystals that can be reliably detected and measured are approximately 13 μm in length.

Although crystals smaller than 13 μm may be visually identifiable, background noise associated with *in situ* acquisition prevents reliable segmentation and quantitative analysis. Therefore, crystals below 13 μm were excluded from number density calculations.

Crystal counting was performed directly on the reconstructed images and slices using ImageJ.

We have added all this information to the Methods section to clarify how crystal number density was determined and to specify the detection limits of the technique (Lines 565-584).

Lines 242–243: As noted above, both nucleation and growth rates were controlled by undercooling. The supposed inverse relationship with superheating is unclear.

We thank the reviewer for this comment. We agree that undercooling is the driving force of nucleation and growth rates. After careful revision, we explicitly acknowledged the role of undercooling and integrated it in the revised text (Lines 264–307; see also reply to comments above for more context).

Lines 739–741: Indicate which experimental run was used for the in-situ data.

We thank the reviewer for this comment. We have now specified in the text which experimental run was used for the *in situ* data (Line 882).

Reviewer #3 (Remarks to the Author):
Dear Editor, Dear Authors,
I have now reviewed the manuscript from Bonechi et al. "The role of superheating on clinopyroxene nucleation delay in mafic magmas: implications for pre-eruptive crystal content and magma ascent dynamics".
The manuscript describes the results of crystal nucleation experiments performed both in-situ and ex-situ, finding that increasing overheating increases the delay for crystal nucleation, which they show impacts on eruptive dynamics.
I think this is a very good study, however there are various issues that have to be addressed.
The most important for me is the framing of the study. When I read the manuscript the first time, I thought almost until the very end that the authors were considering models in which magma is reheated by magma injection above its liquidus temperature, which I think has basically no applications to nature (I will explain later why). I think the problem starts at Lines 60-61 (…."for example through the injection of mafic magma). First, I explain why reheating significantly above liquidus temperature is essentially not possible in nature (especially when the main application of this study is to the 2021 La Palma eruption):
Reheating magma above its liquidus temperature in nature is extremely difficult and thus the "overheating by magma injection" scenario might only apply to the injection of large masses of basalt into a chemically evolved reservoir, which would significantly limit the applications of your study and make it less impactful (which would be a shame). My suggestion would be to frame your experiments to consider decompression-induced overheating
Consider for instance La Palma: magma produced in the mantle is extracted (by definition) at the solidus temperature of the mantle (and the liquidus temperature of the basalt). If this magma is injected in a reservoir of the same magma that has just cooled and crystallised (i.e. the liquidus temperature has not changed if water was not removed from the system), the injection of the same basalt extracted from the mantle cannot results into an increase of temperature above liquidus (as the liquidii of the two magmas are the same). And even to bring back the temperature of the magma in the reservoir to values close to its liquidus temperature, would require the injection of a mass of magma that is much much larger than the mass of magma within the reservoir.
If the resident magma has lost water, but its chemical composition did not evolve significantly, it is not possible to increase the temperature above its liquidus, injecting its parental magma.
Now, I explain why this is not only a semantic problem and reframing the study on decompression induced-overheating requires some attention to avoid internal contradiction of the arguments presented by the authors.
The hypothesis proposed by the authors to explain the impact of overheating on nucleation is summarised at Lines 204-208: "Combined observations from DSC and in situ view experiments conducted near the liquidus suggest that incipient crystalline clusters or nanolites, possibly formed during the initial heating ramp, may act as heterogeneous nucleation sites and significantly reduce incubation times. Conversely, superheating above the liquidus leads to the dissolution of such structures, yielding a more structurally homogeneous and disordered melt. This homogenization increases the thermodynamic and kinetic barriers to nucleation, thereby extending the nucleation delay by requiring greater undercooling to re-establish favourable conditions for crystal formation". If the results of the experiments are framed in this way, the hypothesis stands only if magma is reheated above its liquidus temperature, but it is not granted that this model would apply to overheating occurring during the adiabatic (or quasi-adiabatic) ascent of magma, which does not imply a supply of energy to the system, but actually a decrease of heat associated to the work done for the expansion of the melt to take place.
Something I disagree with. The authors state at Lines 150-152 that: "The different pressures of

the in situ and ex situ view experiments do not significantly affect the comparability of the experimental results, since pressure has a negligible effect on crystallization kinetics". *Actually, your glasses are fully degassed, thus an increase of pressure will result in an increase of liquidus temperature of the material. This means that the liquidus temperature should be higher for the experiments performed in ex-situ conditions with respect to those performed in-situ. Under these conditions, if the initial temperature is the same, the degree of undercooling is lower for the ex-situ experiments than for the in-situ ones. Hammer (2008) describes the effect of undercooling on crystal nucleation delay: (cit. page 35) " Striking contrasts between quasi-continuous decompression and single-step decompression run products underscore the importance of decompression history in controlling crystal texture (Fig. 15). For example, samples decompressed to final pressures >40 MPa in multiple steps were much less likely to reach equilibrium crystallinity than samples decompressed in a single step. Because crystallization during each decompression step brings the system closer to equilibrium, stepwise decompression produces smaller instantaneous* $\Delta Teff$ *than is typical in the single-step decompressions. That the slowly-decompressed samples were less likely to achieve chemical equilibrium than their rapidly-decompressed counterparts is interpreted to reflect this generally lower degree of undercooling in the multistep run throughout the experiment. This observation runs contrary to conventional wisdom, which would suggest slow decompression is more likely to promote chemical equilibration than rapid decompression."*
The multiple steps decompression experiments in Hammer 2008 are equivalent to smaller degrees of undercooling in your experiments (i.e. the ex-situ experiments), and therefore according to Hammer 2008, it is expected that nucleation is delayed in your higher-pressure experiments. Perhaps this is something the authors can use to reframe their study?

We thank the reviewer for the insightful and constructive comments. Answers to all comments can be found in the following replies.

Comments from the PDF
Lines 56-57: This sentence follows the first and should state clearly why crystal content is important for "changing eruptive style". One way would be the impact of crystal content on magma viscosity. Please specify in the text.

We thank the reviewer for this helpful comment. We have revised the text to explicitly clarify why crystal content is a key parameter in controlling eruptive style, by highlighting its first-order influence on magma viscosity and on the efficiency of degassing, both of which strongly affect eruptive behaviour (Lines 58-60).

Lines 60-61: What about superheating resulting from decompression of water undersaturated magma? This should be consider as the water content of La Palma is sufficiently low that magma would have been overheated for most of its ascent from the lithosphere.

Blundy, J, K V Cashman, A Rust, and F Witham. "A Case for CO2-Rich Arc Magmas." Earth and Planetary Science Letters 290, nos. 3–4 (2010): 289–301.

We thank the reviewer for this comment. We have expanded the discussion of superheating mechanisms to explicitly include decompression-induced overheating of water undersaturated magmas during ascent, which is particularly relevant for mafic systems such as La Palma. In the revised text, we clarify that superheating is considered in a relative sense (i.e., relative to the liquidus temperature), and does not necessarily require external heat input via magma recharge. We also added a reference to Blundy et al. (2010) to support this interpretation (Lines 63–66).

Line 122: Please clarify if you assume magma is cooling upon ascent (i.e. non-adiabatic ascent) or if you consider that the degree of undercooling increases because of the increase of liquidus temperature associate with the release of H2O.

We thank the reviewer for this comment. In the *in situ* experiments, the increase in undercooling is intended to account for both non-adiabatic cooling during ascent and the increase in liquidus temperature associated with volatile exsolution (e.g., H_2O loss) upon decompression. We have revised the text accordingly to explicitly include both processes (Lines 142–145).

Lines 125-126: This is unclear. Are you first increasing the temperature above the cpx liquidus of 88 degrees and then cooling down at constant rate? please modify this sentence. Also, 88 degree is a very precise values, I suggest rounding this number as I do not think we know TLcpx so precisely

We thank the reviewer for this comment. Yes, in the *ex situ* experiments the temperature was first increased to approximately 90 °C above the clinopyroxene liquidus. The system was then cooled at a constant rate of 1 °C min^{-1} until reaching 1110 °C, i.e., just below the clinopyroxene liquidus temperature for the *ex situ* conditions (T_{Lcpx} ~1112 °C). The experiments were subsequently held at this temperature for the duration of the run before the final quenching (Fig. 1). We have clarified this experimental procedure in the revised text (Lines 147–150) and rounded the superheating value to ~90 °C throughout the revised manuscript.

Line 139: ..."maintaining the temperature constant for..."

Done. Lines 162-163.

Line 141: Please specify somewhere if there is a difference between the cpx liquidus temperature estimates with rhyolite melts and with the 4D experiments. I heard multiple times that r-MELTS tends to overestimate liquidus T, but perhaps this is not the case?

We thank the reviewer for this comment. We have clarified in the revised manuscript that the clinopyroxene liquidus temperature estimated with Rhyolite-MELTS (~1,140 °C at 20 MPa) closely matches the value observed during the 4D *in situ* experiments, indicating that the MELTS estimate does not overestimate TL_{cpx} in our system. This clarification has been added in the revised manuscript (Lines 170-172).

Lines 142-144: What do you mean by relaxation? that the glass chips start to loose their original form?

We thank the reviewer for the request for clarification. By "relaxation," we refer to the structural relaxation of the glassy starting material, which occurs as the glass passes its glass transition temperature (T_g). Above T_g, the glass transitions toward a molten liquid state and begins to respond dynamically to temperature, stress, or other perturbations on timescales faster than our observations. This process results in the gradual loss of the original shape of the glass chips and is key to understanding melt behaviour prior to crystallisation. We have clarified this point in the revised manuscript (Lines 167–169).

Lines 150-152: Actually, your glasses are fully degassed, thus an increase of pressure will result in an increase of liquidus temperature. This means that the liquidus temperature should be higher

for the experiments performed in ex-situ conditions with respect to those performed in-situ. Under these conditions, if the initial temperature is the same, the degree of undercooling is lower for the ex-situ experiments than for the in-situ ones. Hammer (2008) describes the effect of undercooling on crystal nucleation delay: (cit. page 35) "Dynamic experiments. Striking contrasts between quasi-continuous decompression and single-step decompression run products underscore the importance of decompression history in controlling crystal texture (Fig. 15). For example, samples decompressed to final pressures >40 MPa in multiple steps were much less likely to reach equilibrium crystallinity than samples decompressed in a single step. Because crystallization during each decompression step brings the system closer to equilibrium, stepwise decompression produces smaller instantaneous ΔTeff than is typical in the single-step decompressions. That the slowly-decompressed samples were less likely to achieve chemical equilibrium than their rapidly-decompressed counterparts is interpreted to reflect this generally lower degree of undercooling in the multistep run throughout the experiment. This observation runs contrary to conventional wisdom, which would suggest slow decompression is more likely to promote chemical equilibration than rapid decompression."

The multiple steps decompression experiments in Hammer 2008 are equivalent to smaller degrees of undercooling in your experiments (i.e. the ex-situ experiments), and therefore according to Hammer 2008, it is expected that nucleation is delayed in your higher pressure experiments.

Hammer, Julia E. "Experimental Studies of the Kinetics and Energetics of Magma Crystallization." Reviews in Mineralogy and Geochemistry 69, no. 1 (2008): 9–59. https://doi.org/10.2138/rmg.2008.69.2.

We thank the reviewer for the comment. We have removed the sentence regarding the influence of pressure on crystallisation kinetics, as it was potentially misleading in the context of our comparison between *in situ* and *ex situ* experiments. The focus of our discussion is on the effects of superheating and undercooling, and pressure differences do not alter the main conclusions.

Lines 204-205: I cannot stop thinking about the difference between the process you investigate here (which is surely an important end-member) and the superheating produced by decompression of water undersaturated magmas. In this second case the temperature does not increase (it is only the liquidus temperature that drops with pressure until water saturation increases; see Blundy et al., 2010 EPSL), but nucleation has also been observed to depend on decompression rates and (e.g. Hammer, 2008):
Hammer, Julia E. "Experimental Studies of the Kinetics and Energetics of Magma Crystallization." Reviews in Mineralogy and Geochemistry 69, no. 1 (2008): 9–59. https://doi.org/10.2138/rmg.2008.69.2.

We thank the reviewer for the comment. We have considered the suggested process and integrated the discussion in the relevant sections of the revised manuscript (see responses to other comments for details).

Lines 213-214: I think for this scenario to make sense in nature, the author should consider that to reheating a magma above its liquidus, requires the injection of a lot of magma and of a different magma with higher liquidus temperature.
Consider for instance La Palma: magma produced in the mantle is extracted (by definition) at the solidus temperature of the mantle. If this magma is injected in a reservoir of the same magma that has just cooled and crystallised (i.e. the liquidus temperature has not changed if water was

not removed from the system), the injection cannot results into an increase of temperature above liquidus. And even to bring back the temperature of the magma in the reservoir to close to its liquidus temperature, would require the injection of a mass of magma that is much much larger than the mass of magma within the reservoir.
If the resident magma has lost water, but its chemical composition did not evolve significantly, it is not possible to increase the temperature above its liquidus, injecting its parental magma. Thus, the overheating by injection scenario might only apply to the injection of large masses of basalt into a chemically evolve reservoir, which however would significantly limit the applications of your study and make it less impactful (which would be a shame). My suggestion would be to frame your experiments to consider decompression-induced overheating

We thank the reviewer for this comment. We agree that superheating can occur via multiple mechanisms in natural magmas, including both magma recharge and decompression of water undersaturated melts during ascent. We have revised the manuscript to explicitly acknowledge these processes (Lines 237–248). In addition, we have added a section at the end of the Discussion (Lines 391–418) and introduced a new figure (Figure 6 and below). This figure compares Rhyolite-MELTS liquidus calculations with P-T paths derived from the 1D conduit model to quantitatively explore how oxygen fugacity, H_2O content, and disequilibrium crystallisation modulate the thermal evolution of ascending mafic magma. Although La Palma is used as a representative case study, the modelling framework is broadly applicable to mafic volcanic systems.

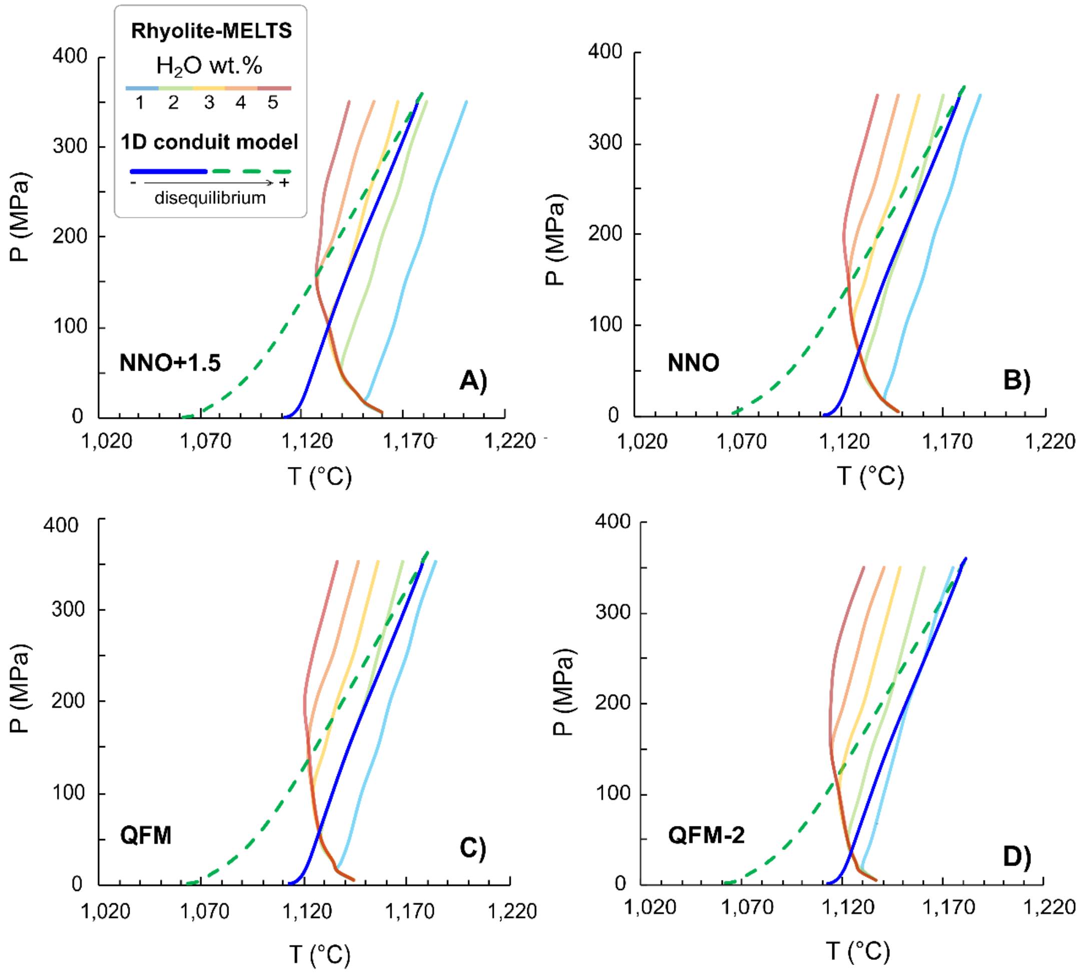

Line 224: This is not surprising if you consider that the liquidus temperature at lower pressure is lower. Because of this in the ex-situ experiments the time available for growth is longer than in the in-situ experiments

We thank the reviewer for this comment. In the revised Discussion section, we have incorporated changes that reflect the suggestions here and those from the other reviewers (Lines 235–307).

Lines 232-236: again similar to Figure 15 of Hammer 2008.

We thank the reviewer for this insightful comment. We agree that the differences in nucleation behaviour between the *in situ* and *ex situ* experiments can also be interpreted in the context of decompression history and effective undercooling, as described by Hammer (2008). In our *ex situ* experiments, the combination of higher pressure and nearly constant undercooling (ΔT_{uc} ~12 °C) results in delayed nucleation, analogous to the reduced nucleation efficiency observed in multi-step decompression experiments. We have integrated this discussion into the revised manuscript to clarify that the lower and nearly constant number of crystals in the *ex situ* experiments reflects both the effect of superheating and the influence of undercooling under higher-pressure conditions (Lines 269–273).

Lines 259-262: YES! I agree. add references.

We added some references in the revised manuscript (Line 323).

Lines 268-273: I cannot agree more

Thanks.

Reviewer #5 (Remarks to the Author):
The manuscript "The role of superheating on clinopyroxene nucleation delay in mafic magmas: implications for pre-eruptive crystal content and magma ascent dynamics" by Bonechi et al. presents an experimental and numerical modeling study on the reheating of magma above the clinopyroxene liquidus, followed by subsequent cooling. The authors argue that such reheating alters crystallization behavior because the dissolution of pre-existing crystals promotes homogeneous crystallization, which operates on significantly longer timescales compared with heterogeneous crystallization. This mechanism has implications for magma ascent and eruption style, as reduced crystallization results in lower melt viscosity and potentially higher ascent velocities.
Overall, this is an interesting and well-written study with a clear structure and a logical flow. The research question is highly relevant, as the pre-eruptive processes governing the effusive–explosive transition, and eruption style more broadly, remain poorly constrained. Any contribution that refines our understanding of such processes is therefore valuable.
I find the experimental component, involving superheating and subsequent crystallization, to be well designed and presented. I have no major comments on that section.
The main concerns I have, relate to magma ascent modeling. The simulations only model the effect of varying crystallization timescales rather than explicitly modeling superheating. Previous studies (e.g., Kozono & Koyaguchi, 2012) have already demonstrated how crystallization kinetics influence eruption style, so the modeling results as presented, do not appear to introduce fundamentally new insights.

We thank the reviewer for the careful reading of the manuscript and for the positive assessment of the experimental component of our study. We also appreciate the constructive comments regarding the magma ascent modelling. Answers to all comments can be found in the following replies.

<u>Specific comments</u>

• *The 2021 Cumbre Vieja eruption has been extensively studied in recent years. I expected a more detailed synthesis of available data (e.g., Ubide et al., 2023; Romero et al., 2022; Dayton et al., 2024, etc) regarding eruption styles (fountaining, effusion, etc.) and their correlation with pre-eruptive temperatures, crystal contents, and related parameters, rather than the broad discussion presented in lines 302–312. Since the authors propose that superheating may have influenced eruption style, the manuscript would benefit from more concrete observational constraints supporting this hypothesis.*

We thank the reviewer for this thoughtful comment and for highlighting the extensive body of work on the 2021 Cumbre Vieja eruption. We agree that the eruption has been thoroughly documented, including detailed reconstructions of eruptive styles, magma compositions, and temporal variations in pre-eruptive conditions. An additional statement has been included in the revised manuscript (Lines 122-127), in addition to the information already reported in the previous version (Lines 375-383). Our objective, however, is not to provide a comprehensive synthesis or reinterpretation of the Cumbre Vieja eruption sequence, nor to quantitatively reconstruct its temporal evolution. Rather, the aim of this study is to investigate a general physical mechanism, namely the effect of superheating-induced nucleation delay on crystallinity, magma viscosity, and ascent dynamics, that may operate broadly in mafic magmatic systems.

We use the 2021 Tajogaite magma as a well-constrained case study because its composition is well characterized and was used as the starting material in our experiments, allowing a direct link between experimental results and realistic magmatic conditions. In this framework, the eruption provides a natural context in which the proposed mechanism may plausibly operate, rather than a system we seek to model explicitly. We have clarified this point in the revised Introduction (Lines 118-130) and Discussion (Lines 339-342).

In addition, we have revised the Discussion section (Lines 335–418) to better emphasize the general applicability of the proposed mechanism to mafic volcanic systems, with La Palma serving as a representative and well-documented example rather than the primary focus of the investigation.

.• *The study focuses on clinopyroxene crystallization. While clinopyroxene was indeed the dominant crystalline phase during the 2021 eruption, plagioclase, Fe–Ti oxides, and amphibole were also present. How are these phases treated—or neglected—in the simulations, and what are the implications of excluding them?*

We thank the reviewer for this important point. In the numerical simulations, crystallisation is not treated on a phase-by-phase basis. Instead, the model tracks the total crystal fraction in the magma, which is the primary parameter controlling rheology (through viscosity) and, consequently, magma ascent dynamics. Clinopyroxene is central to this framework because it is the first major phase to crystallise in the investigated tephritic magma and therefore determines the onset and early increase of crystallinity during cooling and ascent. The experimentally observed nucleation delay of clinopyroxene following superheating thus provides a conservative and physically meaningful proxy for the timing of crystallisation onset. Delaying clinopyroxene nucleation effectively delays the development of bulk crystallinity and its rheological impact.

The crystallisation model itself is derived from a fit to results obtained from over 100,000 Rhyolite-MELTS simulations performed across a range of pressures, temperatures, and H_2O contents.

MELTS accounts for the formation of all crystalline phases in the system; in our approach, we extract the total crystal fraction from these simulations to construct the model. Therefore, while the simulations track bulk crystallinity rather than individual phases explicitly, all phases are implicitly included through their contribution to the total crystal content.

Other crystalline phases observed during the 2021 eruption, such as plagioclase, Fe–Ti oxides, and amphibole, typically crystallise at lower temperatures and/or at later stages of ascent. Their contribution is therefore expected to mainly influence the later evolution of crystallinity, after the critical rheological threshold controlling early ascent dynamics has already been established.

We have clarified this modelling assumption in the revised Supplementary Data, explicitly stating that the simulations account for bulk crystallinity rather than individual mineral phases, and that experimentally measured clinopyroxene nucleation delay is used as a representative proxy for the onset of crystallisation in mafic magmas.

• *If one simulation case is intended to represent superheating, why are the initial temperatures identical in both cases? Similarly, why is the initial crystal content the same? Superheating should imply partial or complete dissolution of crystals prior to ascent, so why the same initial crystal content (and temperature) for both?. Although the authors state that the key factor is the kinetic slowdown caused by melt homogenization, the conduit simulations reproduce results already shown in previous works (e.g., Arzilli et al., 2019), without adding something new regarding the different consequences of superheating.*

We understand the point of the reviewer. Below, we provide a clearer description of the scenarios simulated by the conduit model.

Let's consider a magma which is coming from a deeper source than our conduit inlet (in our case > 13 km depth) at superheated conditions, and let's assume that this magma is entering a reservoir (just below the inlet of the conduit) which is at near-liquidus conditions for one crystal phase with a given volume fraction of phenocrysts of other crystal phases. As demonstrated by our experiments, depending on the degree of superheating, nucleation delay may vary from tens of minutes up to several hours, which means that the fresh magma will cool down without crystallising for a certain amount of time.

With our numerical simulations we want to consider two end-member cases. One end-member case assumes that the fresh superheated magma rapidly enters the reservoir at 13 km, cools to liquidus conditions, and entrains phenocrysts from the resident magma before entering the conduit for the final ascent to the surface. In this case, as a consequence of this rapid transit, the residence time in the reservoir is negligible compared to the nucleation delay, so we can assume that nucleation delay is still the same as that at superheated conditions (8 hours, as shown by the experiments at high degree of superheating). The other end-member case assumes that the fresh superheated magma circulates and cools down within the reservoir at near liquidus conditions for a timescale much longer than the nucleation delay. In this case, being the temperature of the magma chamber at near-liquidus conditions, nucleation delay during ascent can be assumed 20 minutes (as shown by the experiments at near liquidus conditions).

Numerical simulations obtained assuming a nucleation delay of 8 hours show that magma ascent time is ~30 minutes and therefore syn-eruptive crystallisation is completely inhibited, producing a fountaining eruption. On the other hand, assuming a nucleation delay of 20 minutes, syn-eruptive crystallisation does occur within the conduit. As a result, viscosity increases, slowing down magma ascent even further and allowing more time to crystallise. This generates a positive feedback, which eventually produces an effusive eruption.

We understand that these scenarios represent extreme cases, but they are instructive because they tell us that the pre-eruptive history of magma can have a key role on magma ascent dynamics. Furthermore, to appreciate the effect of different nucleation delays on magma ascent dynamics, it is important to compare results starting from the same thermodynamic conditions.

As suggested by the reviewer, we can perform numerical simulations with different initial conditions, simulating a hotter magma with very low phenocryst content and a colder magma with a given volume fraction of phenocryst. However, how can we distinguish between the role of the initial conditions compared to the effect of superheating on nucleation delays in this way?

Following the suggestion of the reviewer, we reported below numerical results obtained assuming 1) a nucleation delay of 8 h, an inlet temperature of 1180 °C and an initial phenocryst content of ~3 vol.%; and 2) a nucleation delay of 20 min, an inlet temperature of 1150 °C and an initial phenocryst content of ~27 vol.%.

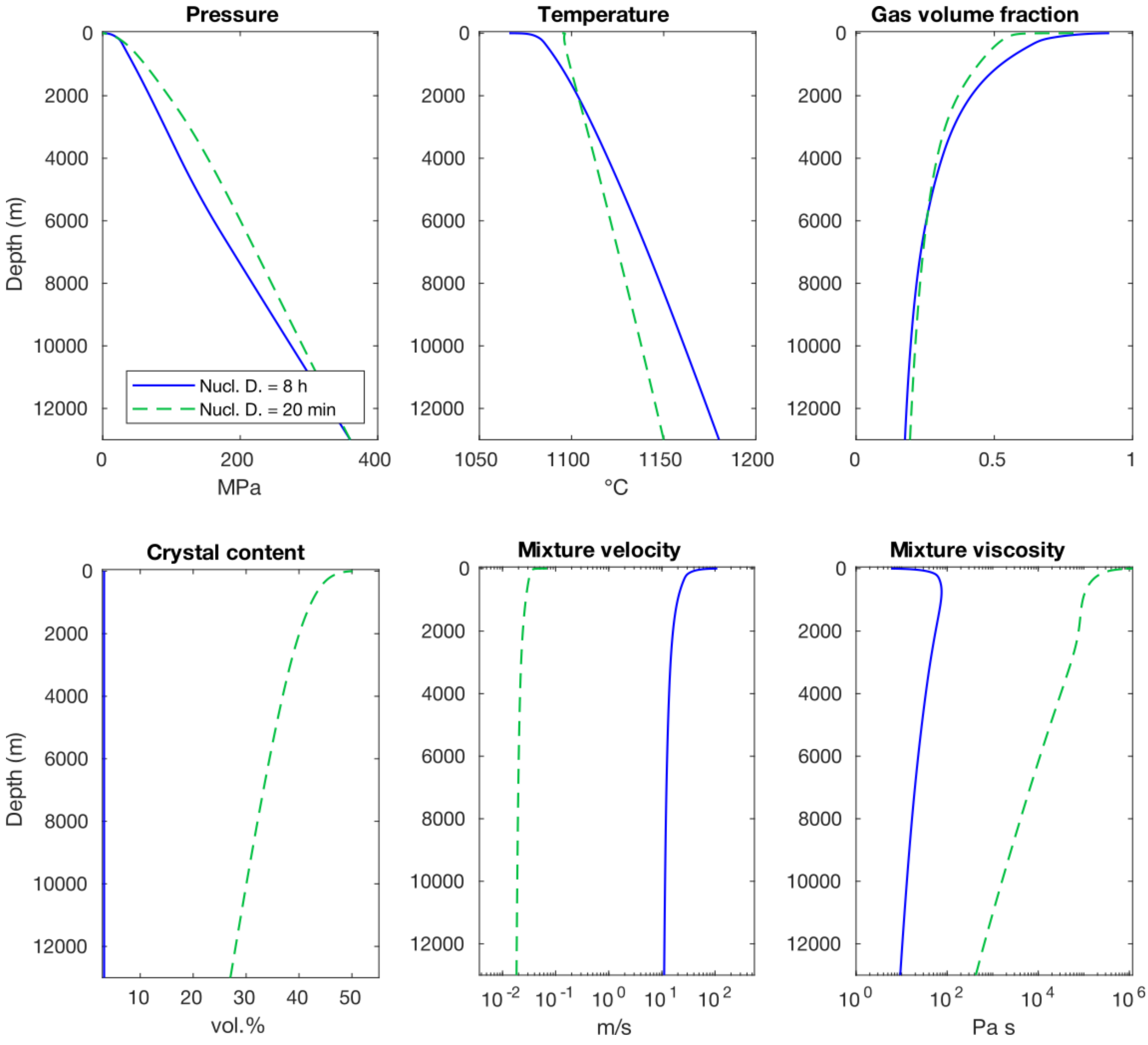

The numerical simulations and the style of the activity are clearly different. But is this difference related to the nucleation delay, or rather the initial conditions? To answer this question, we compared numerical simulations with the same initial conditions and different nucleation delays, and results are plotted below:

1) an inlet temperature of 1180 °C and an initial phenocryst content of ~3 vol.%:

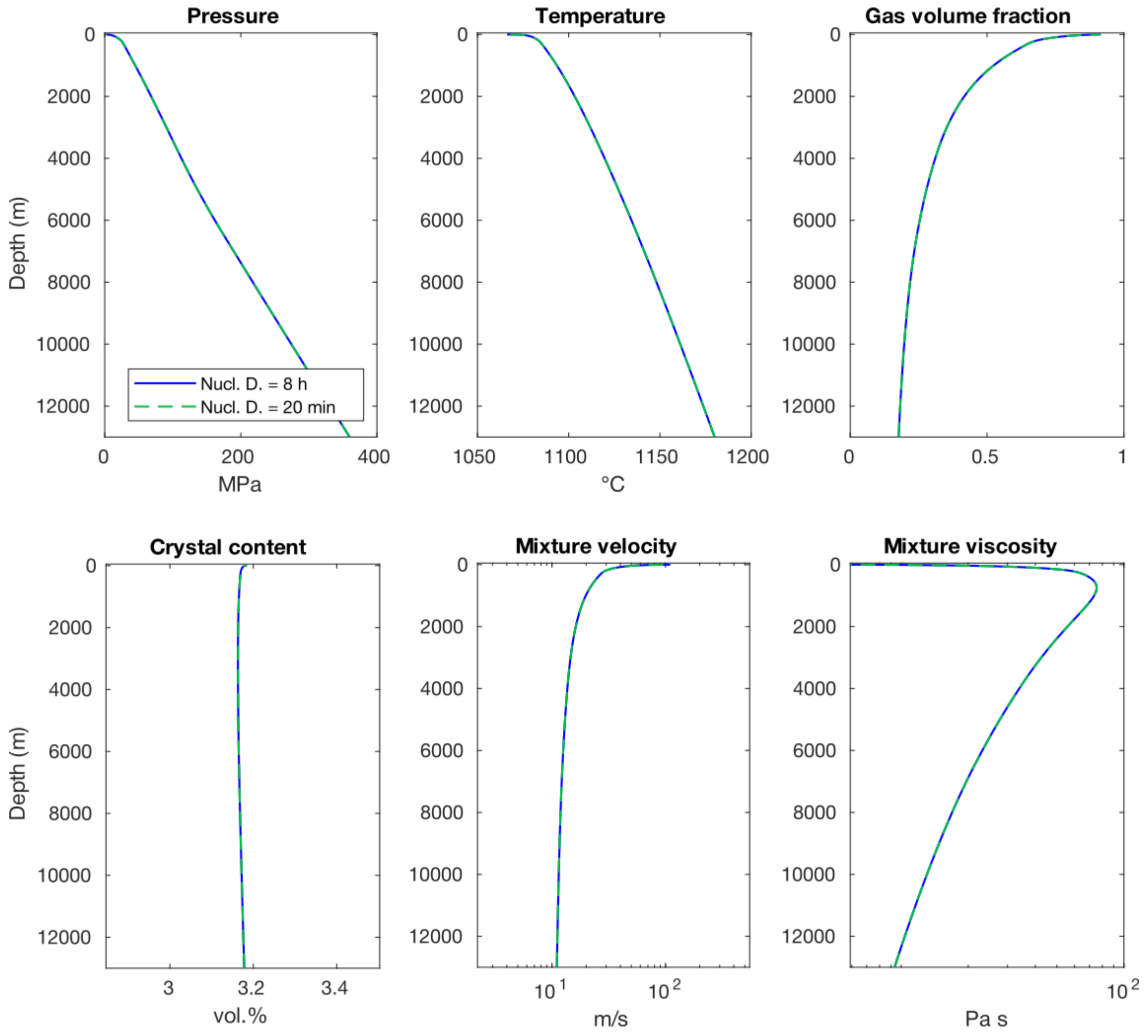

2) an inlet temperature of 1150 °C and an initial phenocryst content of ~ 27 vol.%:

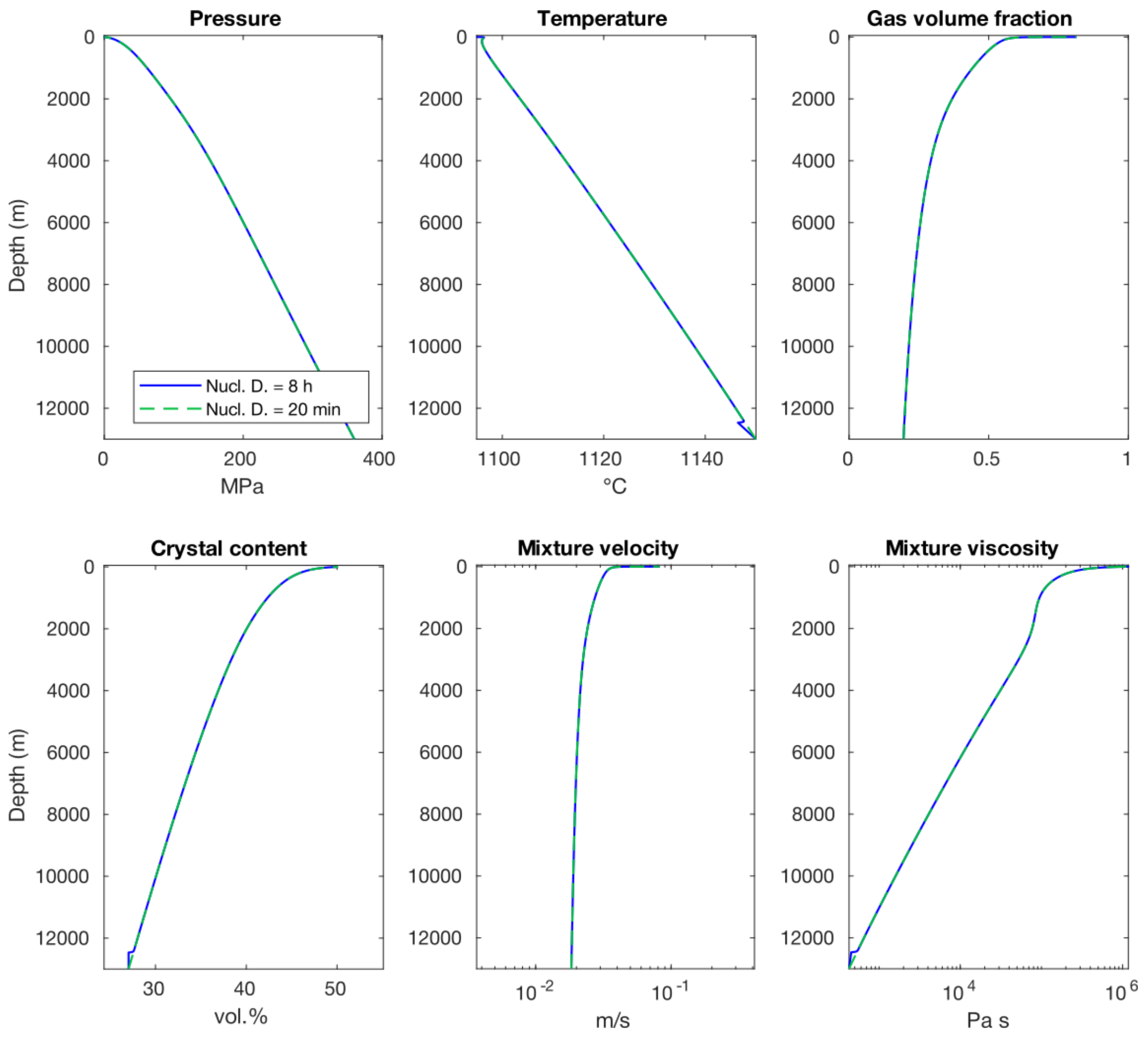

As we can see, notwithstanding the different nucleation delay, the profile of the variables along the conduit are basically the same. This is because the pre-eruptive thermodynamic conditions play a primary role in controlling magma ascent dynamics. Indeed, if magma ascent time is shorter than 20 minutes, crystallisation will be inhibited in any case due to the fast ascent. Similarly, if magma ascent time is much longer than 8 hours, even if crystallisation will start shallower in the conduit, eventually it will reach the equilibrium value, resulting in very similar profiles. The role of nucleation delay becomes critical when the ascent time is comparable with the nucleation delays. This has been now clarified in the revised text (Lines 370–373).

<u>*Other comments*</u>

• *In the title and subtitles, the phrase "… implications for pre-eruptive crystal content" could be misleading, as it may be interpreted as referring to initial crystal content in the reservoir. A more accurate phrasing might be "implications for ascent crystallization."*

We thank the reviewer for this comment. In our study, "pre-eruptive crystal content" refers specifically to the crystal content in the magma chamber prior to eruption. Superheating and nucleation delay in the chamber directly influence this crystal content, which in turn controls

magma rheology, ascent dynamics, and eruptive style (Lines 313–318). While these processes also affect crystallisation during ascent, which is why we included the conduit model, our experiments do not provide direct information on ascent crystallisation. Therefore, we focus on the crystal content established within the reservoir.

• *Figures 3 and 4 could be moved to the Supplementary Material, as they are not essential for the main narrative*.

We thank the reviewer for the suggestion. We have moved the figures to the Supplementary Materials and adjusted the figure order accordingly.

Conclusion

In summary, this is a potentially strong manuscript that merits publication. However, the section on implications for ascent dynamics requires further refinement. In particular, demonstrating how superheating influences ascent dynamics not only in crystallization kinetics but also in initial crystal content and temperature, and incorporating more detailed observational data from the 2021 Cumbre Vieja eruption that supports the main hypothesis, would substantially strengthen the authors' conclusions.

We thank the reviewer for this positive evaluation of our manuscript. The issues highlighted in the concluding remarks have been addressed in detail in our responses to the previous comments. In particular, we now summarise the conditions and scenarios under which superheating can influence conduit dynamics and present the new results obtained from the conduit model. The manuscript has been revised accordingly, including modifications to the text and the addition of two new figures to clarify and strengthen these aspects.

Response to reviewers' comments for NCOMMS-25-57465A

Responses to reviewers' comments and changes in the main text are reported in light blue

Reviewer #2 (Remarks to the Author):
This is my second review of the manuscript. I am satisfied with the way my comments and those of the other reviewers have been addressed. I have no further comments and recommend acceptance of the manuscript.

We thank the reviewer for the positive assessment and for confirming that all previous concerns have been satisfactorily addressed. We are pleased that the manuscript is now suitable for publication.

Reviewer #3 (Remarks to the Author):
Dear Authors,
I appreciate the effort to perform the calculations linked to the new Figure 6 and I think this could potentially be a nice addition to the paper.
However, in the current format the results are not correctly presented and discussed.
At lines 411-415 the authors state: "Our results indicate that superliquidus conditions are favoured under more reduced conditions and at higher H2O contents, as liquidus temperatures decrease under these conditions. The modelled scenarios here define distinct superheating regimes governed by different degrees of disequilibrium crystallisation during ascent, with liquidus positions modulated by water content and redox state". The issue here is that the initial temperature of the P-T path is always the same. This is incorrect as the magma liquidus' temperature is linked to its water content and initial oxygen fugacity (initial because the oxygen fugacity of magmas will change upon ascent, see for example Burgisser and Scaillet , 2007 Nature). For instance, if a magma contains more water its initial liquidus temperature will be lower so the initial point of the curves at lower temperatures should be lower. Both curves in Figure 6 will always lead to crystallisation because their slope is lower than the slope of the liquidii for magmas with different water contents.
Water content and (potentially) oxygen fugacity will impact the evolution of the magma's crystal content only if they affect the slope of the liquidus curves with respect to the slope of the P-T path considered. I urge the authors to take care of this section and Figure 6.

We thank the reviewer for this insightful comment. Following this feedback, we sought clarification through direct discussion with the reviewer, which helped us better understand the concern regarding the dependence of liquidus temperature on initial H_2O content and oxygen fugacity, and the implications for the P–T paths shown in Figure 6.
We agree that, as currently formulated, the figure does not adequately capture these dependencies, particularly the shift in initial liquidus temperatures under varying volatile contents and redox conditions. As a result, the representation may be misleading in terms of the relative slopes of the P–T paths and liquidus curves, and the associated crystallisation behaviour.

As Figure 6 was introduced only as a schematic addition in response to a previous comment and is not central to the main results of this study, we have decided to remove it and the corresponding discussion section to avoid potential misinterpretation.
Importantly, the key conceptual aspects raised by the reviewer have been incorporated into the revised manuscript (Lines 323-331), where we now more clearly discuss the role of H_2O content and redox conditions in controlling liquidus temperatures and magma evolution, as well as superheating during adiabatic decompression.

Reviewer #5 (Remarks to the Author):
I thank the authors for the substantial revision of the manuscript, "The role of superheating on clinopyroxene nucleation delay in mafic magmas: Implications for pre-eruptive crystal content and magma ascent dynamics." Overall, I find the revised version significantly improved relative to the original submission. In the first round, my main concerns were focused primarily on the magma ascent modelling rather than on the experimental component, which I already considered strong. In this revised version, the modelling section is clearer, better motivated, and more closely connected to the experimental results than before.
Most importantly, the authors have now modified the conduit model to account explicitly for nucleation delay, rather than relying only on contrasting crystallisation timescales. This is a meaningful improvement and addresses one of my main concerns from the previous round. The revised manuscript now uses nucleation delays derived from the experiments (20 min versus 8 h) as end-member inputs in the modelling framework, which provides a much more direct conceptual connection between the experiments and the numerical exploration.
I also appreciate that the revised manuscript now makes much clearer that the purpose of the modelling is not to reconstruct the 2021 Tajogaite eruption in detail, but rather to use Tajogaite as a representative mafic case study to explore a more general physical mechanism. This clarification improves the framing substantially. In the original version, the application to Tajogaite felt too specific relative to the level of constraint provided by the model. The revised text is more appropriately framed in this respect.
The discussion of other crystalline phases is also improved. The authors now explain more explicitly that the simulations track total crystal fraction rather than phase-specific crystallisation, and that clinopyroxene is treated as a representative proxy for the onset of crystallisation because it is the dominant early phase in this system and the one constrained experimentally. I find this acceptable within the scope of the study.
That said, I still have one remaining concern, which is mostly about emphasis and interpretation, rather than about the core validity of the study. Even in the revised version, the modelling remains an illustrative, end-member-based exploration, not a quantitatively constrained reconstruction of Tajogaite. I therefore encourage the authors to keep the interpretation carefully phrased throughout, especially where the manuscript suggests that superheating may explain specific eruptive transitions or variations in eruptive style during the 2021 eruption. In my view, the study now demonstrates a plausible and potentially important mechanism, but not yet a uniquely constrained explanation for Tajogaite itself. Related to this point, I think the manuscript would benefit from one final pass to ensure that the strongest claims are consistently aligned with what the model actually shows. The real strength of the study is that it demonstrates how experimentally motivated nucleation delays can have major consequences for ascent dynamics under certain initial conditions and timescales. This is already a valuable result. It does not need to be overstated as a specific explanation for the natural evolution of Tajogaite. The authors themselves now acknowledge that pre-eruptive thermodynamic conditions primarily control magma ascent dynamics, and that nucleation delay becomes particularly important when ascent timescales are comparable to nucleation-delay timescales. I agree with that formulation and suggest that it should guide the tone of the final discussion.

Recommendation
Overall, I consider that the authors have addressed my main concerns, and that the revised manuscript is now suitable for publication after minor revision.
Remaining minor points
I suggest that the authors strengthen the discussion of the Tajogaite case study by briefly reporting the

observed range of eruptive or mass discharge rates during the 2021 eruption, and clarifying whether the modelled contrast between the two end-member scenarios is comparable in magnitude to the natural variations documented during Tajogaite. This would provide a more concrete basis for evaluating whether the proposed role of superheating-induced nucleation delay is consistent with the scale of variability observed in nature. At present, the manuscript suggests that superheating may have contributed to the variability in eruptive style and intensity, but this link would be more convincing if the authors explicitly compared the modelled changes with the natural range of eruptive-rate variability inferred for Tajogaite.

We thank the reviewer for the thorough and highly constructive assessment of the revised manuscript, and for the positive evaluation of the improvements made to the modelling framework and overall framing. We are particularly pleased that the reviewer finds the revised interpretation more appropriately balanced and better connected to the experimental constraints.

We also appreciate the remaining suggestion regarding the comparison between model outputs and the observed variability in eruptive or mass discharge rates during the 2021 Tajogaite eruption. This is an important point that further strengthens the link between the modelling results and natural observations.

In response, we have added a brief discussion in the revised manuscript reporting the observed range of eruptive/mass discharge rates documented for the 2021 eruption and explicitly comparing these values with the variability produced by the two end-member model scenarios (Lines 378–387). This addition highlights that the modelled differences fall within the same order of magnitude as the natural variability, and helps to better constrain the interpretative scope of the model.

Finally, we have carefully revised the relevant sections of the Discussion (Lines 311–404) to ensure that all interpretations remain fully consistent with the illustrative, end-member nature of the modelling approach. In particular, we now explicitly emphasise that the results demonstrate a physically plausible mechanism controlling ascent dynamics under specific timescale conditions, and are not intended to provide a uniquely constrained reconstruction of the temporal evolution of the Tajogaite eruption.
